# Supplementary material for: A Resident Morbidity and Mortality Conference Curriculum to Teach Identification of Cognitive Biases, Errors, and Debiasing Strategies
Source: MedEdPORTAL. 2021 Oct 28;17:11190. doi: 10.15766/mep_2374-8265.11190 (PMC8551265; doi:10.15766/mep_2374-8265.11190)
Supplement: Supplementary file 1 — M&M Resident Presenter Guide.docxM&M Advisors Guide.docxM&M Introduction and Template.pptxM&M Discussion Handout.docx [file mep_2374-8265.11190-s001.zip › B. M&M Advisors Guide.docx]

**Morbidity and Mortality Advisor’s Guide**

Below are guidelines for the individual or team taking leadership over the Morbidity and Mortality (M&M) conference series. While chief residents and fellows can help play these roles, we strongly recommend a single faculty advisor take a main leadership role throughout the series.

**Establishing Tone**

Establishing a tone of openness, honesty, and non-punitive self-examination is key to the success of the series. We suggest this be done in multiple ways:

1. Presentation of the introductory session by the faculty advisor, which should include an “M&M” presentation of the advisors own case:
   1. The case should be one in which the advisor was actively involved in patient care, and in which the advisor can identify cognitive errors and bias.
   2. The case should be presented in the same manner expected of the residents, including open discussion with the audience about the cognitive errors and biases present.
   3. The advisor should emphasize lessons learned, debiasing strategies implemented, and coping strategies used in-order to emphasize the importance of this kind of work beyond residency, throughout a physician’s career.
2. Explicit “ground rules” presented with each M&M presentation, including the introductory session:
   1. The discussion should remain private, and not discussed outside the setting of M&M conference.
   2. The contents of M&M are “non-discoverable” and therefore legally protective (though this is state dependent, so state and hospital policies should be investigated by the faculty advisor
   3. The tone of the session is non-judgmental, non-punitive, and always respectful.
3. Moderate discussion during the presentation and discussion:
   1. Redirect or correct comments made by the audience, or the presenter, that do not adhere to the above “ground rules.”
   2. Communicate with those who persistently violate ground rules outside the M&M setting. This is particularly important if faculty/attendings are in violation.
   3. Thank presenters for their openness in sharing.

**Scheduling**
Residents should be aware of their assigned presentation date several months in advance whenever possible. This allows for adequate time to identify an appropriate case/cases. Program leadership should be involved in ensuring assignments are made with consideration given to the residents’ schedules and anticipated workload.

**Assisting in Case Selection:**

Resident presenters will be expected to share a brief description of the case they have selected for M&M presentation 1 month prior to scheduled presentation. Your role in approving a case is in:

- Determining if the resident had adequate participation in the case to be able to speak to their own individual cognitive biases and errors, as well as those of the team.
- Determining if true *preventable* morbidity or mortality, rather than inevitable disease progression. In some cases, a near miss, with potential for significant morbidity or mortality, may be acceptable.
- Assessing the resident’s emotional readiness to present a case. This is especially important in recent cases, cases of significant personal error, and/or cases involving mortality

**Assisting in Resident Presentation Preparation:**

- Help residents in identifying specific faculty mentors for the case
  - May need help in approaching mentors in particularly sensitive cases or in identifying alternate mentors when resident select a mentor who is unable or unwilling to participate
  - The best mentors were themselves involved with the case
- Review presentation slides 1 week prior to presentation
  - Provide feedback related to presentation aesthetics and flow
    - Can include feedback on wordiness of slides, use of visuals
  - Provide feedback related to content
    - Help residents steer clear of “didactic” style slides
    - Ensure “sensitive” information, especially that related to the role played and errors made by others on the medical team, is presented in a respectful, professional manner, or omitted if this is not possible
  - Assist residents in understanding potential cognitive biases and errors
    - Provide descriptions or clarification of the definitions of individual types of bias
    - Ask questions about specific parts of the case to prompt further reflection
    - Try not to suggest specific biases and errors to the resident in favor of allowing them to self-identify
  - Help residents anticipate what discussion points they may encounter during the presentation
  - Identify and address emotional needs of the resident presenter potentially apparent in the presentation slides.

**Deciding the Appropriate Audience**

- Audience should include resident peers.
- Faculty participation can be very valuable, provided faculty are capable of adhering to ground rules and maintaining the desired tone.
- Participation of medical students may be allowable, depending on circumstances.
- Strictly limit, or prohibit, participation of visitors from outside the institution.

**Moderation of M&M Conference**

- Consistently attend M&M conference to maintain an open tone and develop trust from the resident presenters and audience.
- Ensure “ground rules” are re-iterated with each session.
- Ensure there is a discussion of cognitive errors and biases at the designated time during each presentation.
  - Ensure the audience has the handout (Appendix D) containing brief descriptions of potential cognitive bias.
  - Residents presenters may choose to lead this themselves, but if they prefer, advisor or other leaders (such as chief residents) can do this.
  - Advisor may need to ask questions to encourage audience reflection, but should avoid identifying biases they see themselves (at least until the open discussion at the end of the case)
  - The audience may be able to maintain this discussion relatively independently as they get used the structure.
- Continue to moderate discussion during the “open discussion” section at the end of each case
  - Ensure discussion maintains appropriate tone
  - Offer debiasing strategies or other pearls not previously identified by the presenter or audience

**Follow up**

- Follow up can vary depending on the case and the individual presenter.
  - Avoid “grading” the M&M in-order to reduce anxiety and loss of open tone surrounding M&M.
  - Thank resident for presenting and sharing.
  - Offer further advice and insight to help the resident learn from the case.
  - Connect resident with further resources for coping if needed.
