# Supplementary material for: A Resident Morbidity and Mortality Conference Curriculum to Teach Identification of Cognitive Biases, Errors, and Debiasing Strategies
Source: MedEdPORTAL. 2021 Oct 28;17:11190. doi: 10.15766/mep_2374-8265.11190 (PMC8551265; doi:10.15766/mep_2374-8265.11190)
Supplement: Supplementary file 1 — M&M Resident Presenter Guide.docxM&M Advisors Guide.docxM&M Introduction and Template.pptxM&M Discussion Handout.docx [file mep_2374-8265.11190-s001.zip › D. M&M Discussion Handout.docx]

**Please use the examples below while identifying potential types of cognitive error and bias in this case for discussion with the group:**

Aggregate bias: The tendency to believe that aggregated data, such as those used to develop clinical practice guidelines, do not apply to an individual patient, rooted in the belief the patient is atypical or somehow exceptional.

Anchoring: The tendency to lock onto a diagnosis based on information available early in the patient’s initial presentation, and failure to adjust this initial impression in the light of later information.

Ascertainment bias: Occurs when a physician’s thinking is shaped by prior expectation, preconceived notions, and/or stereotypes.

Availability: The disposition to judge things as being more likely, or frequently occurring, if they readily come to mind. Examples include common, recently encountered, serious, or otherwise noteworthy diagnoses.

Base-rate neglect: The tendency to ignore the true prevalence of a disease, either falsely inflating or reducing its base-rate.

Blind Obedience: Undue deference to authority, technology, or expert.

Commission bias: The tendency towards action, rooted in the principle of beneficence and the belief that harm to the patient can only be prevented by active intervention.

Confirmation bias: The tendency to look for confirming evidence to support a diagnosis rather than look for disconfirming evidence to refute it, despite the latter often being more persuasive and definitive.

Diagnosis momentum: Once diagnostic labels are attached to patients, they tend to become stickier and stickier during subsequent encounters.

Feedback sanction: Making a diagnostic error may carry no immediate consequences, as considerable time may elapse before the error is discovered, if ever, or poor system feedback processes prevent important information on decisions getting back to the decision maker.

Framing effect: How diagnosticians see things may be strongly influenced by the way in which the problem is framed. In terms of diagnosis, physicians should be aware of how patients, nurses, and other physicians frame potential outcomes and contingencies of the clinical problem to them.

Fundamental attribution error: The tendency to be judgmental and blame patients for their illnesses (dispositional causes) rather than examine the circumstances (situational factors) that might have been responsible.

Gambler’s fallacy: Attributed to gamblers, this fallacy is the belief that if a coin is tossed ten times and is heads each time, the 11th toss has a greater chance of being tails. The perceived pretest probability that a patient will have a particular diagnosis might be influenced by preceding but independent events.

Gender bias: The tendency to believe that gender is a determining factor in the probability of diagnosis of a particular disease when no such pathophysiological basis exists.

Hindsight bias: Knowing the outcome may profoundly influence the perception of past events and prevent a realistic appraisal of what actually occurred.

Multiple alternatives bias: The tendency to revert to a smaller subset of possibilities when a multiplicity of options on a differential diagnosis leads to significant conflict and uncertainty, leading to diagnoses not in that subset to be missed.

Omission bias: The tendency toward inaction. Largely this is rooted in the principle of nonmaleficence.

Order effects: Influence the order with which information is obtained or presented has on decision making leading to a failure to give due consideration to all information available.

Outcome bias: The tendency to opt for diagnostic decisions that will lead to good outcomes, rather than those associated with bad outcomes, leading to the perceived probability more serious diagnoses being minimized.

Overconfidence bias: A universal tendency to believe we know more than we do.

Playing the odds: The tendency in equivocal or unclear presentations to opt for a benign or less serious diagnosis on the basis that it is significantly more likely than a serious one.

Posterior probability error: Estimate for the likelihood of disease is unduly influenced by what has gone on before for a particular patient. For example, patients with migraines are more likely to have each headache they present with diagnosed as migraine.

Premature closure: Tendency to accepting a diagnosis before it has been fully verified.

Psych-out error: The vulnerability of patients with psychiatric conditions to errors in management; in particular, comorbid medical conditions may be overlooked or minimized.

Racial bias: The tendency to believe that race is a determining factor in the probability of diagnosis of a particular disease when no such pathophysiological basis exists.

Representativeness restraint: The tendency to look for for prototypical manifestations of disease, which may lead to atypical manifestations being missed.

Search satisfying: The tendency to call off a search once something is found.

Sutton’s slip: The diagnostic strategy of going for the most obvious possibility, in which possibilities other than the obvious are not given sufficient consideration.

Sunk costs: The more clinicians invest in a particular diagnosis (time, resources), the less likely they may be to release it and consider alternatives.

Triage cueing: The influence of “triage,” either within the health system, or “self-triage” of the patient presenting to a specialist or clinical setting, on clinical thinking and decision making.

Unpacking principle: Failure to elicit all relevant information, often in the form of limited or directed history taking.

Vertical line failure: Routine, repetitive tasks often lead to thinking in silos where predictable, orthodox styles that emphasize economy, efficacy, and utility, which can lead to inflexibility and may miss unexpected, rare, or esoteric diagnoses

Visceral bias: The influence of personal feelings towards a patient or their family, either positive or negative.

Yin-Yang out: The tendency to believe that, after an exhaustive and unrevealing workup, nothing further can be done to uncover any definitive diagnosis resides for the patient

**References**

Croskerry P. The importance of cognitive errors in diagnosis and strategies to minimize them. *Academic medicine*. 2003;78(8):775-780.

Ogdie AR, Reilly JB, Pang WG, et al. Seen Through Their Eyes: Residents’ Reflections on the Cognitive and Contextual Components of Diagnostic Errors in Medicine. *Acad Med*. 2012;87(10):1361-1367.
