# Supplementary material for: A Resident Morbidity and Mortality Conference Curriculum to Teach Identification of Cognitive Biases, Errors, and Debiasing Strategies
Source: MedEdPORTAL. 2021 Oct 28;17:11190. doi: 10.15766/mep_2374-8265.11190 (PMC8551265; doi:10.15766/mep_2374-8265.11190)
Supplement: Supplementary file 1 — M&M Resident Presenter Guide.docxM&M Advisors Guide.docxM&M Introduction and Template.pptxM&M Discussion Handout.docx [file mep_2374-8265.11190-s001.zip › A. M&M Resident Presenter Guide.docx]

**Resident Guidelines for M&M Conference**

Morbidity and Mortality (M&M) conference is a forum designed to track and discuss medical error in an environment that facilitates learning, encourages accountability, and promotes leadership and academic development. Goals of M&M are to foster openness in medical error disclosure, facilitate learning from our mistakes, identify and plan to address system issues that permit error. The goal of M&M is NOT to penalize individuals for mistakes or poor outcomes, learn defensive medicine, or mock or second guess management of patients from outside hospitals. Overall, M&M serves an invaluable role in your education and understanding the personal responsibility of a physician to improve patient safety and quality of care at both individual and institutional levels.

**Case Selection:**

The M&M case presentation is intended to be an audience participation conference. Cases are discussed to improve critical decision-making skills. Cases with difficult decisions, complications, or death can be the best cases for all of us to learn from. This conference is not intended to publicly humiliate you for a mistake you have made. Instead, it is a way we all learn from our mistakes, so we improve our care for future patients.

An appropriate case does not require a serious patient outcome or death.

A good case *DOES* require that you were directly involved with the challenges, biases, and/or errors that lead to a less than ideal outcome or near miss. We want to hear about *your* experience.

Cases to consider:

• Diagnostic challenges

• Treatment dilemmas

• Untoward or unanticipated side effects of tests or therapies we employ

• Situations in which the work environment contributed to medical error

• Communication difficulties and miscommunications

• A near misses: an act or decision that could have harmed the patient but did not, also often referred to as “an error that did not reach the patient”

**Case Preparation:**

Consider the following questions about the case before you prepare the case.

**• What** made you **select** this case? (interest, mistake, great save)

• From your case, what is the most important **point** (commit to ONE) you want the audience to remember?

• What were the **critical decisions/dilemmas** during this case?

- Which **biases** affected this patient’s course and care?

• What are the other important or interesting points about this case?

• What is the one thing you would **do differently** next time?

You are encouraged to talk with faculty and other providers involved with the case during your preparation.

**Steps and Guidelines for preparing your M&M:**

- Contact the faculty adviser **at least 1 month** prior to your M&M date to review case selection and provide synopsis of case. (Attach completed “M&M Overview” below.)
- Identify a mentor or mentors for the case **at least 1 month** prior, discuss your case and components that make it an M&M, as well as invite them to come to your M&M presentation.
- Contact the faculty advisor **at least 1 to 2 weeks** prior to your M&M date to review slides.
- Content
  - Briefly discuss the patient’s history and physical followed by timed outline of patient’s clinical course and any studies or interventions.
  - Discuss pertinent errors and biases that influenced the care of the patient:
    - Focus on **cognitive errors and bias.** Review Croskerry article and identify biases and errors that most relate to the case
      - You should include these in your presentation slides, but plan to pause for audience discussion before you present the biases and errors you identified yourself.
    - Also comment on any:
      - Communication issues: written, verbal, with patient, family, providers, or consultants
      - Knowledge base issues
      - Systems issues
      - Teamwork issues: failures, could improvement here have prevented the situation?
      - Equipment issues
      - Procedural complications
  - Be ready to address areas for future improvement, learning points and projected impact on your professional development.
  - Use the attached Power Point presentation (Appendix ***) as a template, but feel free to make modifications as needed to better fit your case.
  - Timing
  - Case should take approximately 15 minutes to present. A lot of the learning in M&M comes from discussion, and we want to budget plenty of time to discuss!
  - Be prepared for two periods of time for discussion during your case.
    - First, after the details of your case are presented, there will be time for audience discussion of cognitive errors and biases during the case.
    - Second, after you finish presenting your content, there should be time for further, more open discussion of any points salient to the case.
  - You should **not** include a didactic component (e.g., you do not need to teach us about a disease process)
  - Consider inviting expert(s) from other specialties to attend, outside of your chosen mentor

**•** Limit PowerPoint slides for the entire presentation.

- - Use slides as prompts
  - Build into presentation prompts to get the audience involved. Use slides to be explicit about EEG, radiographic, laboratory or physical findings. Do not assume audience sees the obvious. Allow them to commit to an interpretation, THEN (or even later) go back and point out the abnormalities or interpretation.

**M&M Timeline**

At least 1 Month prior:

Provide M&M Overview to faculty advisor and your identified Mentor(s)

Invite mentor(s) to M&M Conference for your presentation

1 week prior:

Email presentation to faculty advisor and your identified Mentor(s)

Make any final edits to presentation based on feedback and discussions

Remind mentor(s) of your presentation date and time

**M&M Overview**

** To be emailed to faculty advisor at least 1 month prior to presentation. **

Resident Name:

Date of M&M Presentation:

Mentor(s) Involved:

Synopsis of Case to be Presented:
