# Supplementary material for: A Resident Morbidity and Mortality Conference Curriculum to Teach Identification of Cognitive Biases, Errors, and Debiasing Strategies
Source: MedEdPORTAL. 2021 Oct 28;17:11190. doi: 10.15766/mep_2374-8265.11190 (PMC8551265; doi:10.15766/mep_2374-8265.11190)
Supplement: Supplementary file 1 — M&M Resident Presenter Guide.docxM&M Advisors Guide.docxM&M Introduction and Template.pptxM&M Discussion Handout.docx [file mep_2374-8265.11190-s001.zip › C. M&M Introduction and Template.pptx]

## Slide 1
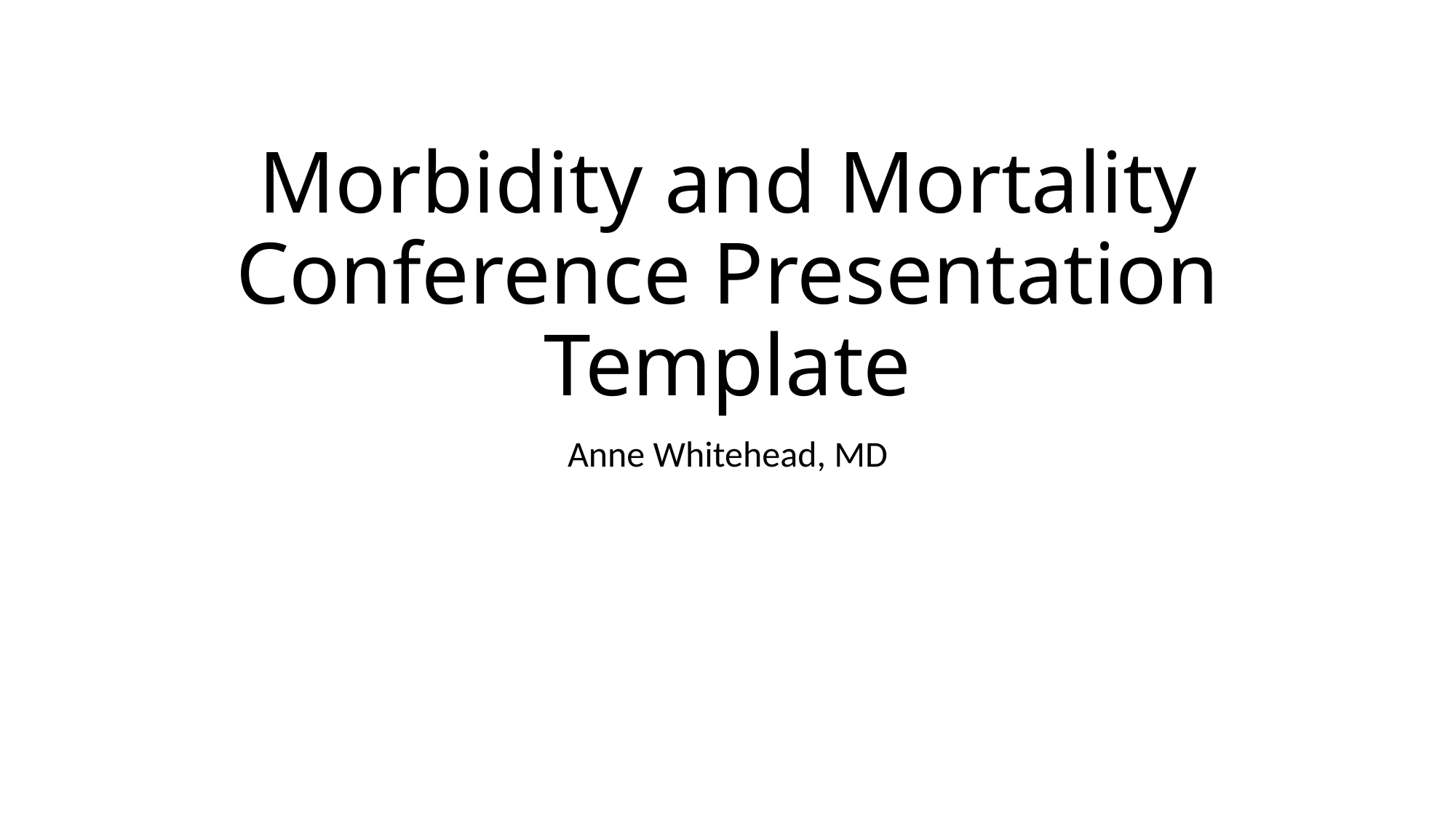

# Morbidity and Mortality Conference Presentation Template
Anne Whitehead, MD

## Slide 2
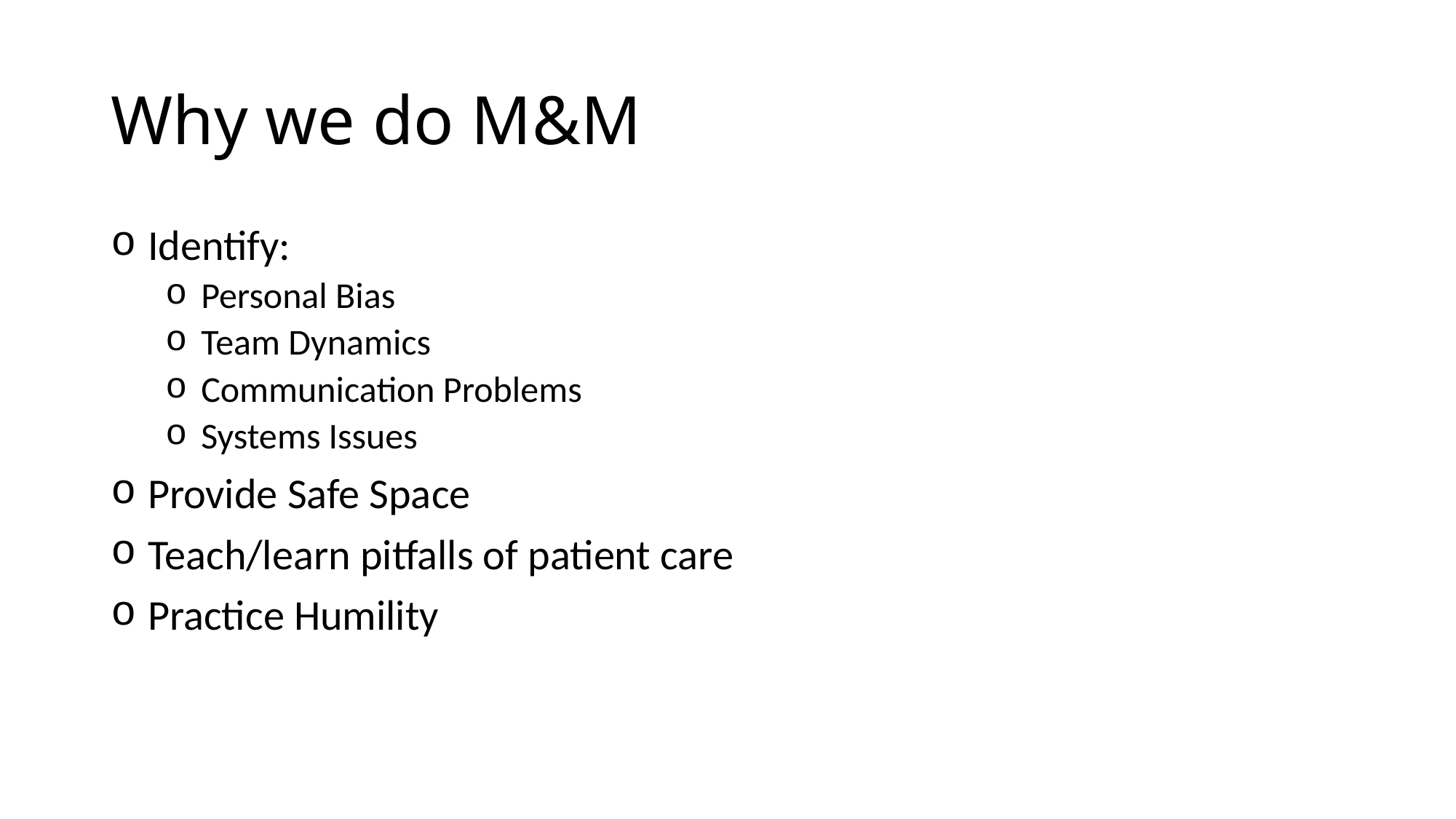

# Why we do M&M
 Identify:
 Personal Bias
 Team Dynamics
 Communication Problems
 Systems Issues
 Provide Safe Space
 Teach/learn pitfalls of patient care
 Practice Humility

## Slide 3
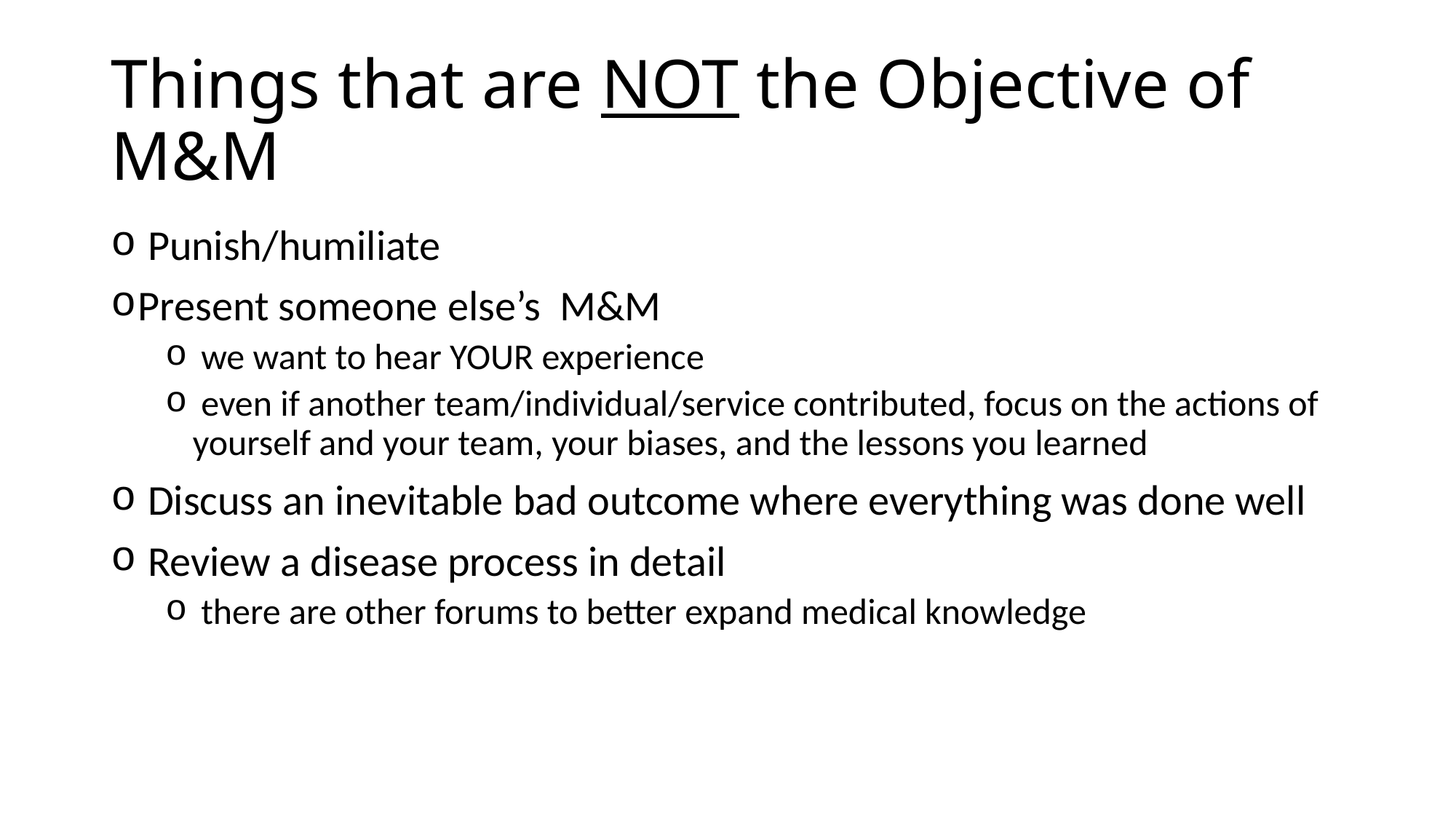

# Things that are NOT the Objective of M&M
 Punish/humiliate
Present someone else’s M&M
 we want to hear YOUR experience
 even if another team/individual/service contributed, focus on the actions of yourself and your team, your biases, and the lessons you learned
 Discuss an inevitable bad outcome where everything was done well
 Review a disease process in detail
 there are other forums to better expand medical knowledge

## Slide 4
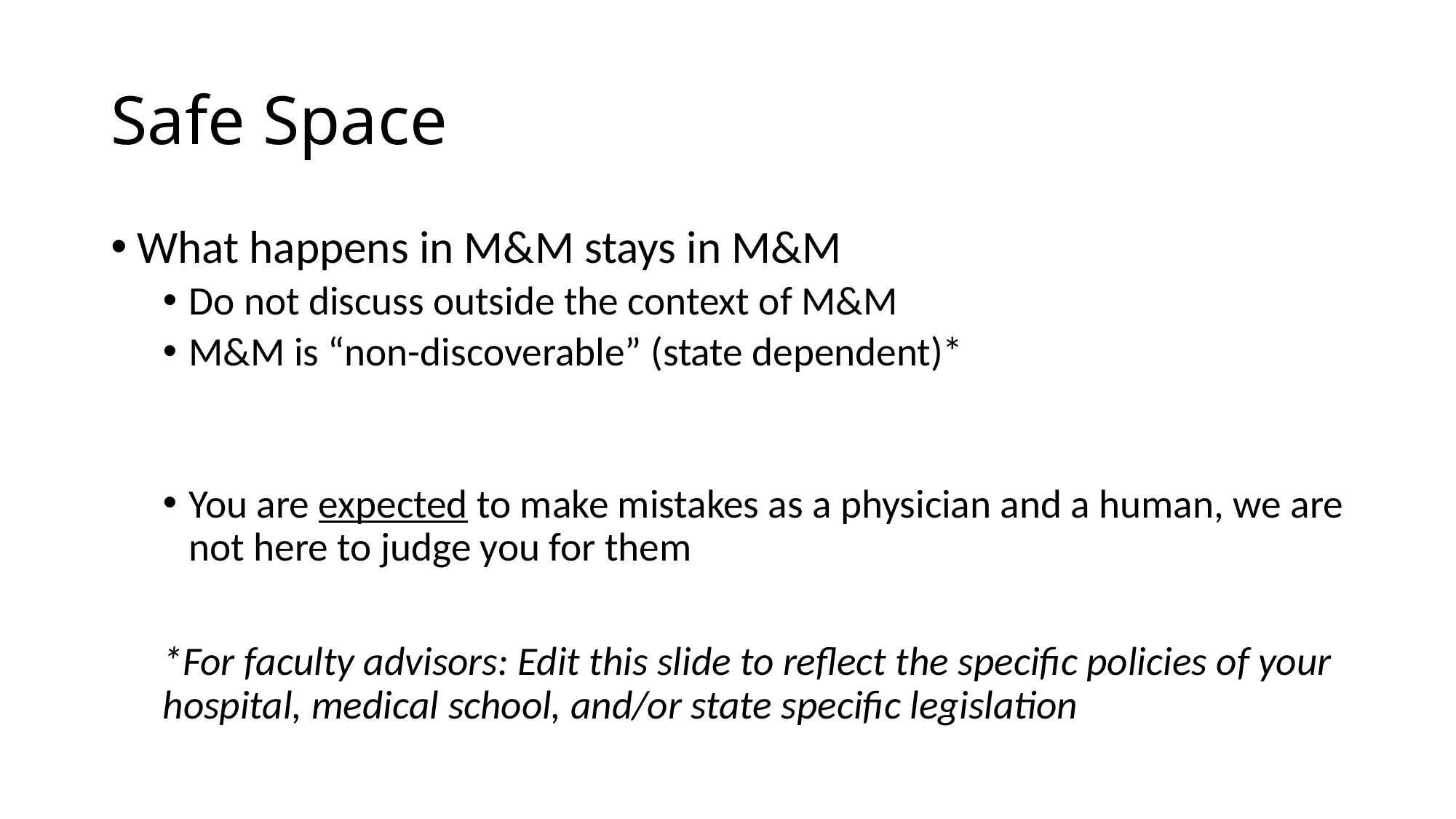

# Safe Space
What happens in M&M stays in M&M
Do not discuss outside the context of M&M
M&M is “non-discoverable” (state dependent)*
You are expected to make mistakes as a physician and a human, we are not here to judge you for them
*For faculty advisors: Edit this slide to reflect the specific policies of your hospital, medical school, and/or state specific legislation

## Slide 5
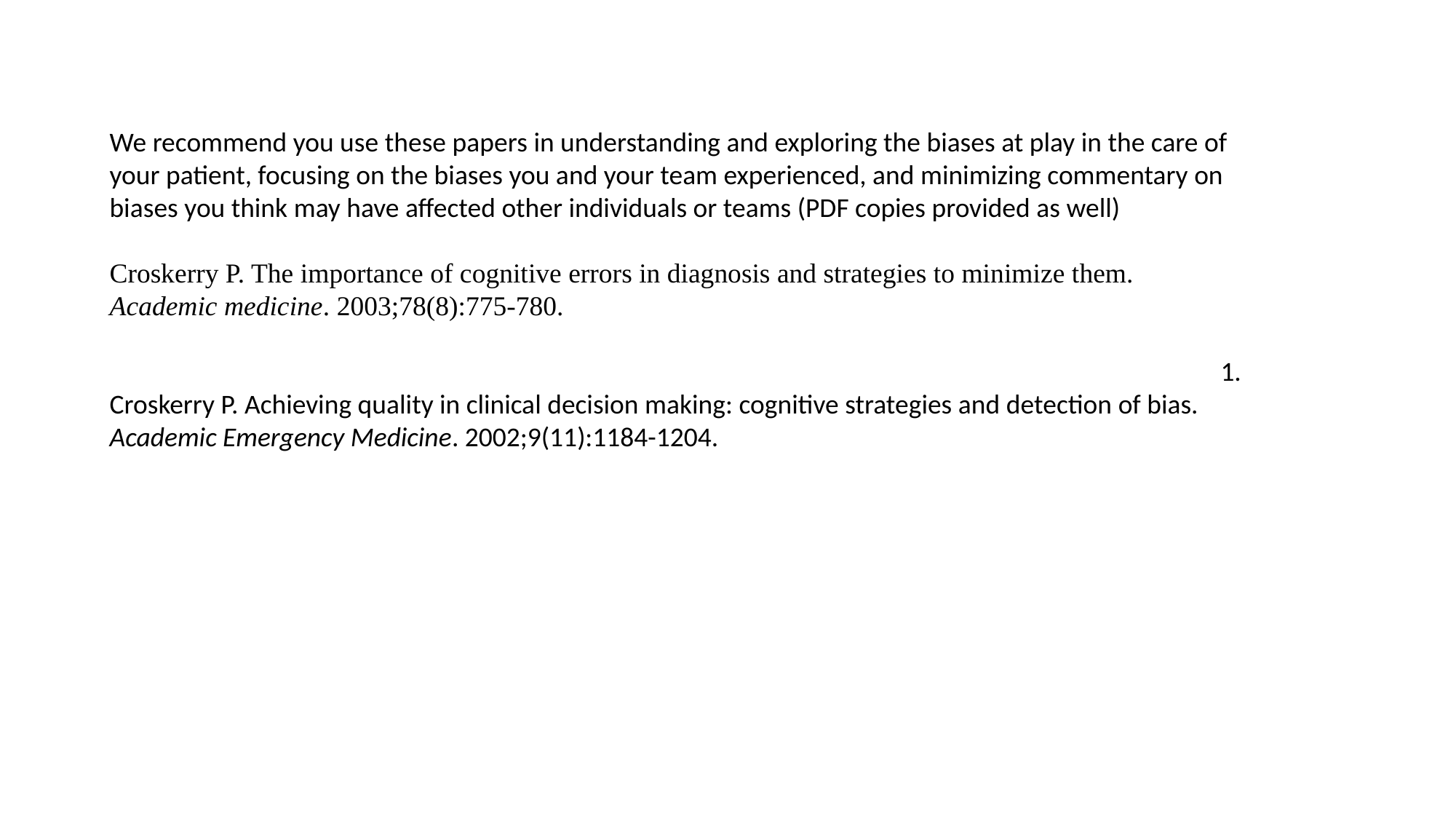

We recommend you use these papers in understanding and exploring the biases at play in the care of your patient, focusing on the biases you and your team experienced, and minimizing commentary on biases you think may have affected other individuals or teams (PDF copies provided as well)
Croskerry P. The importance of cognitive errors in diagnosis and strategies to minimize them. Academic medicine. 2003;78(8):775-780.
1.
Croskerry P. Achieving quality in clinical decision making: cognitive strategies and detection of bias. Academic Emergency Medicine. 2002;9(11):1184-1204.

## Slide 6
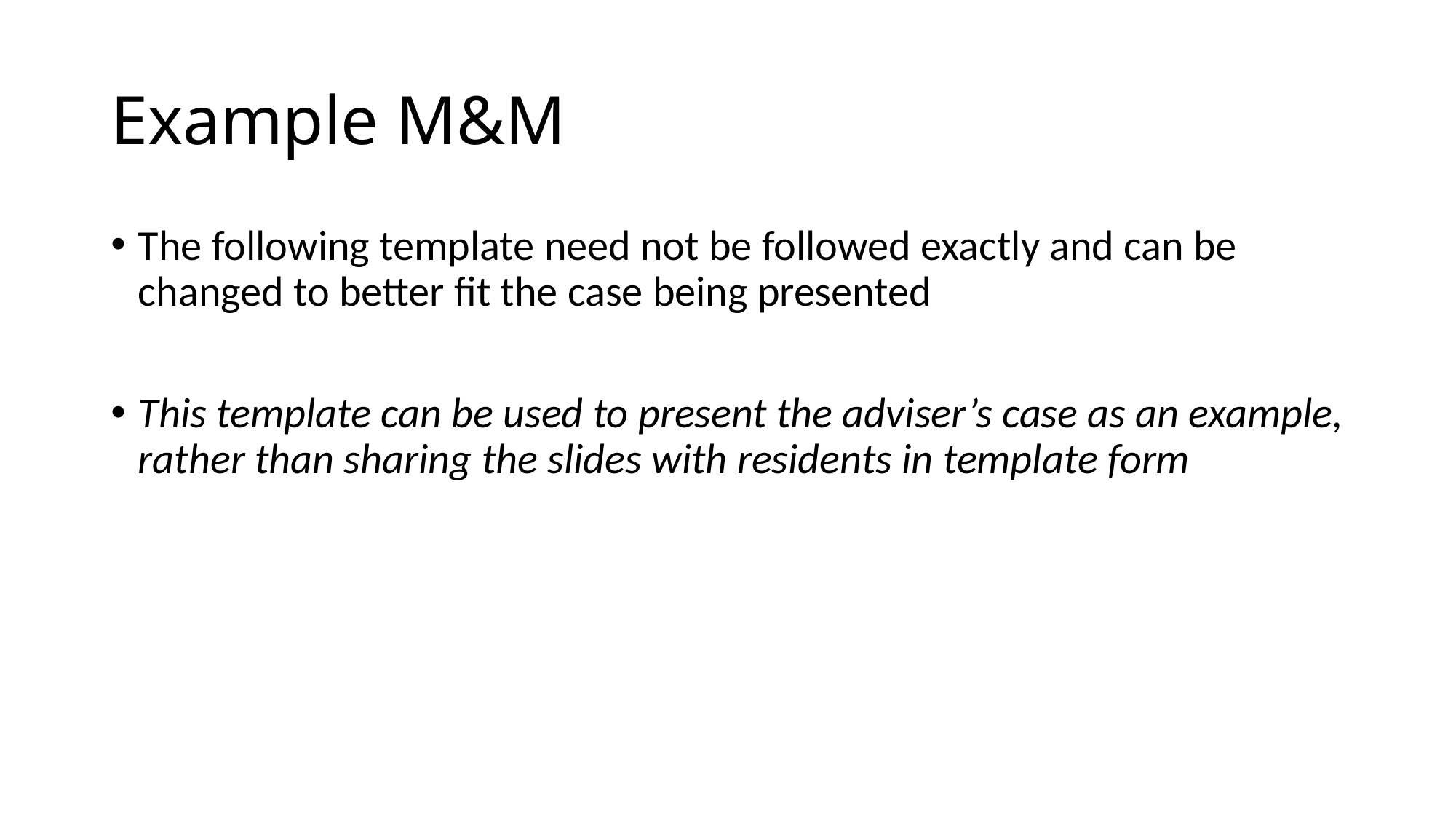

# Example M&M
The following template need not be followed exactly and can be changed to better fit the case being presented
This template can be used to present the adviser’s case as an example, rather than sharing the slides with residents in template form

## Slide 7
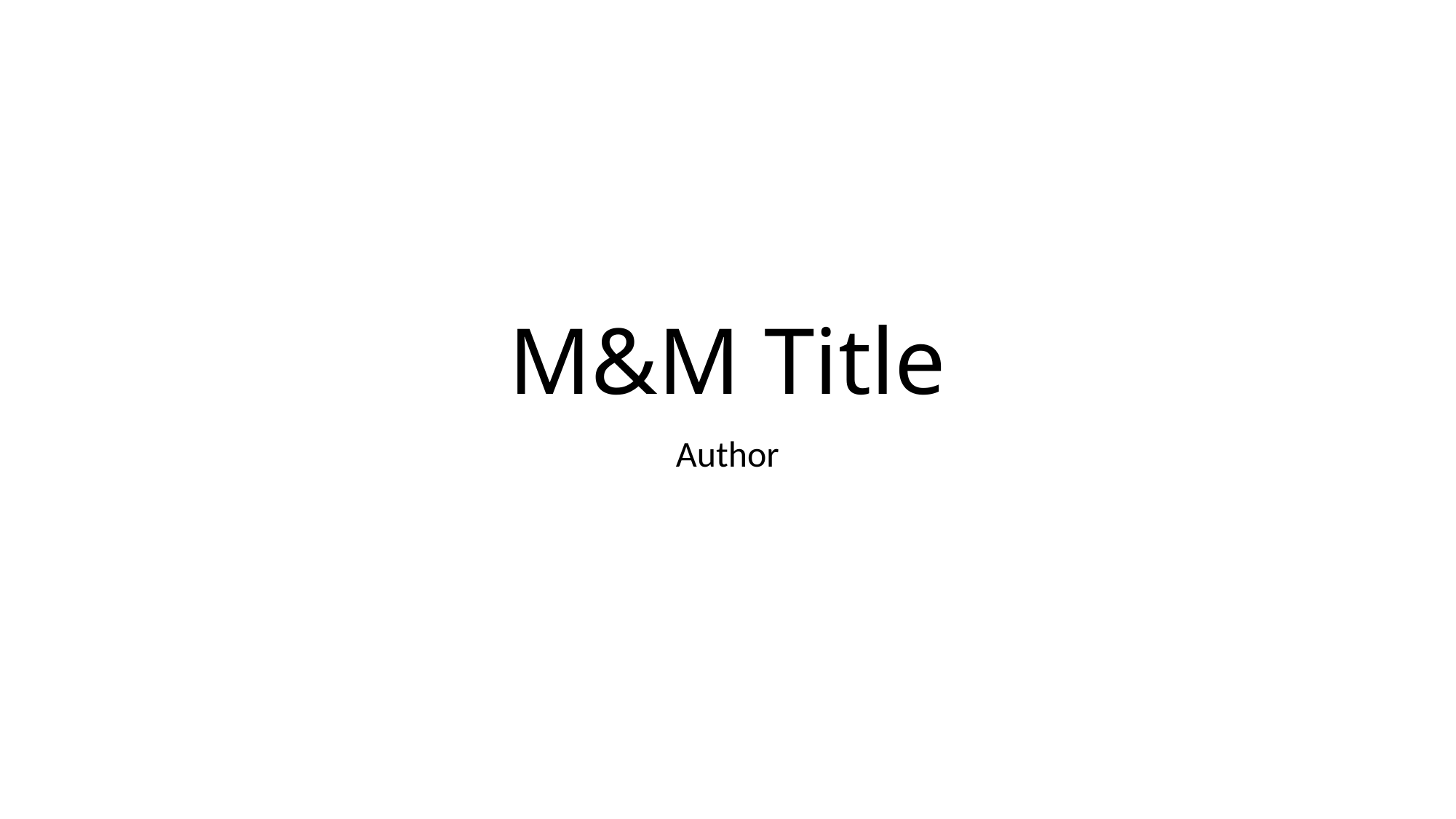

# M&M Title
Author

## Slide 8
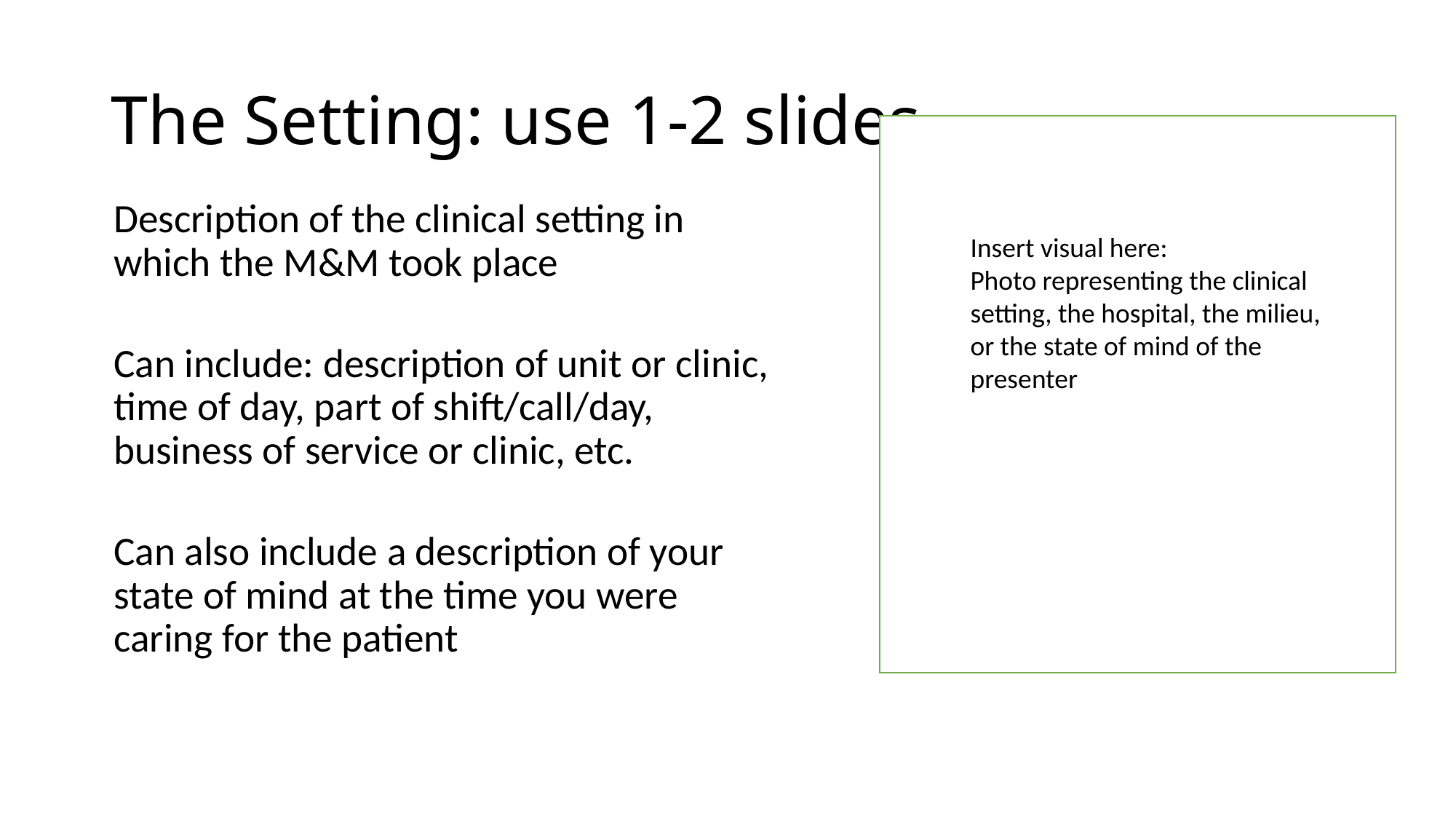

# The Setting: use 1-2 slides
Description of the clinical setting in which the M&M took place
Can include: description of unit or clinic, time of day, part of shift/call/day, business of service or clinic, etc.
Can also include a description of your state of mind at the time you were caring for the patient
Insert visual here:
Photo representing the clinical setting, the hospital, the milieu, or the state of mind of the presenter

## Slide 9
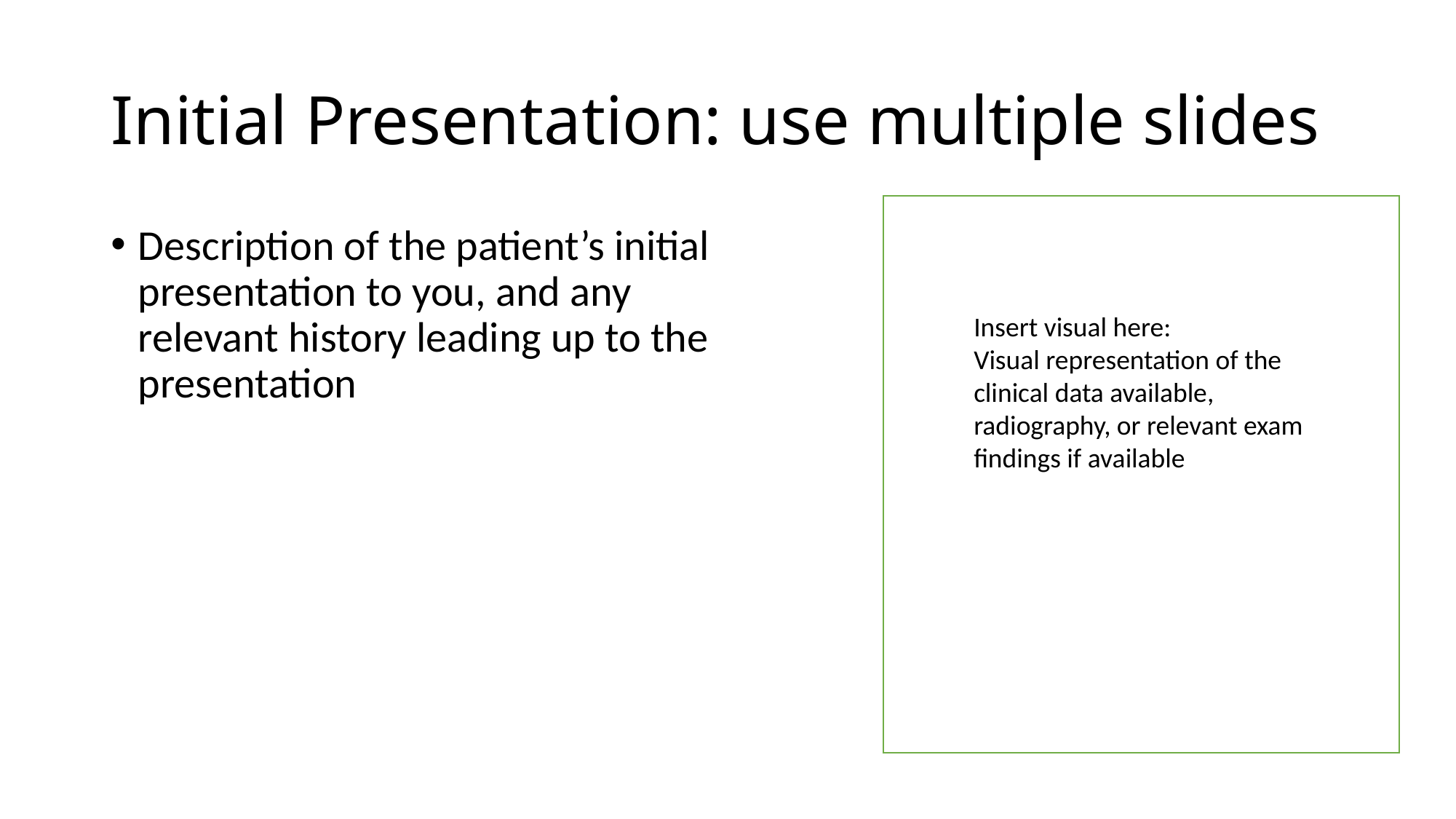

# Initial Presentation: use multiple slides
Description of the patient’s initial presentation to you, and any relevant history leading up to the presentation
Insert visual here:
Visual representation of the clinical data available, radiography, or relevant exam findings if available

## Slide 10
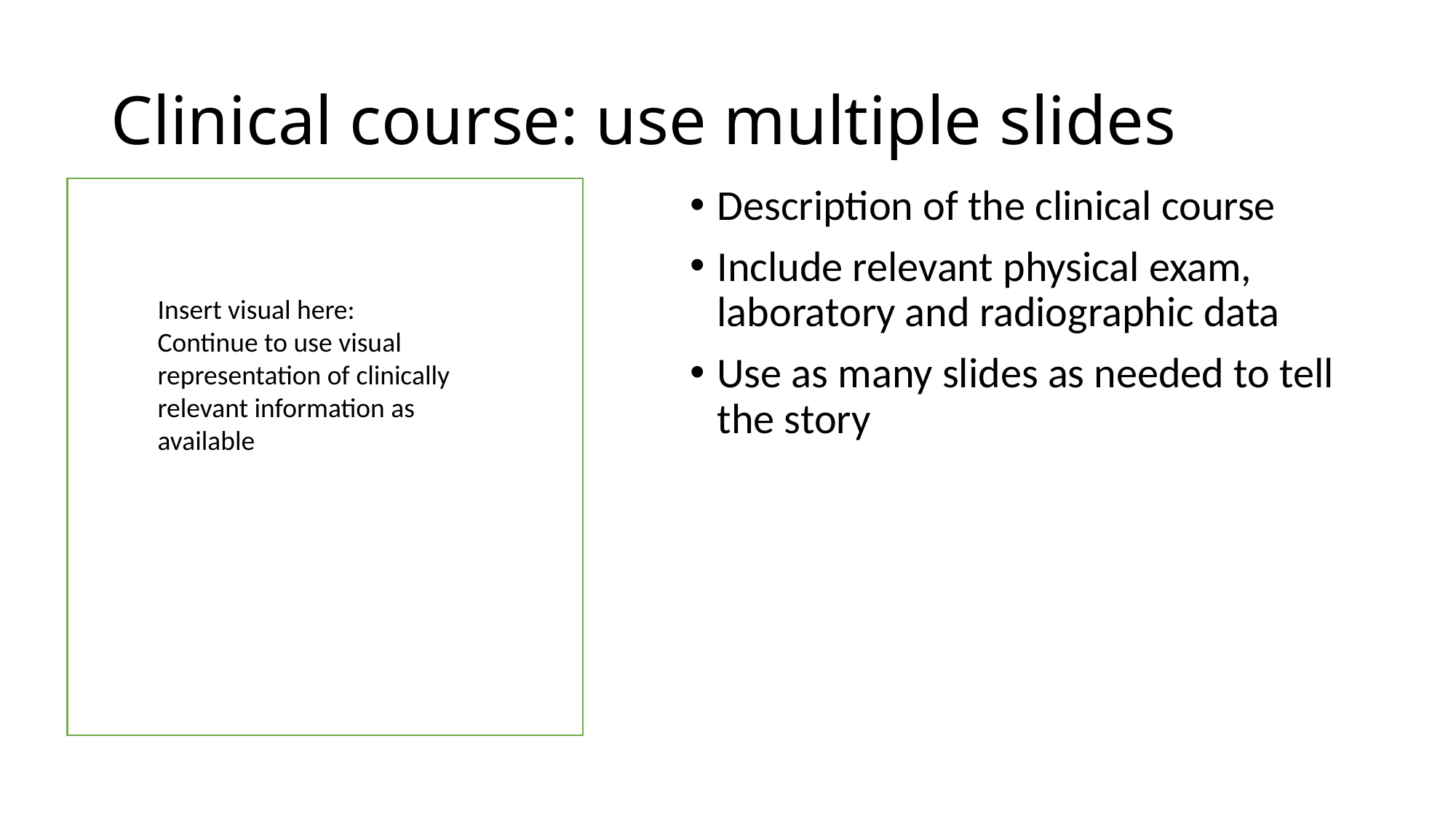

# Clinical course: use multiple slides
Description of the clinical course
Include relevant physical exam, laboratory and radiographic data
Use as many slides as needed to tell the story
Insert visual here:
Continue to use visual representation of clinically relevant information as available

## Slide 11
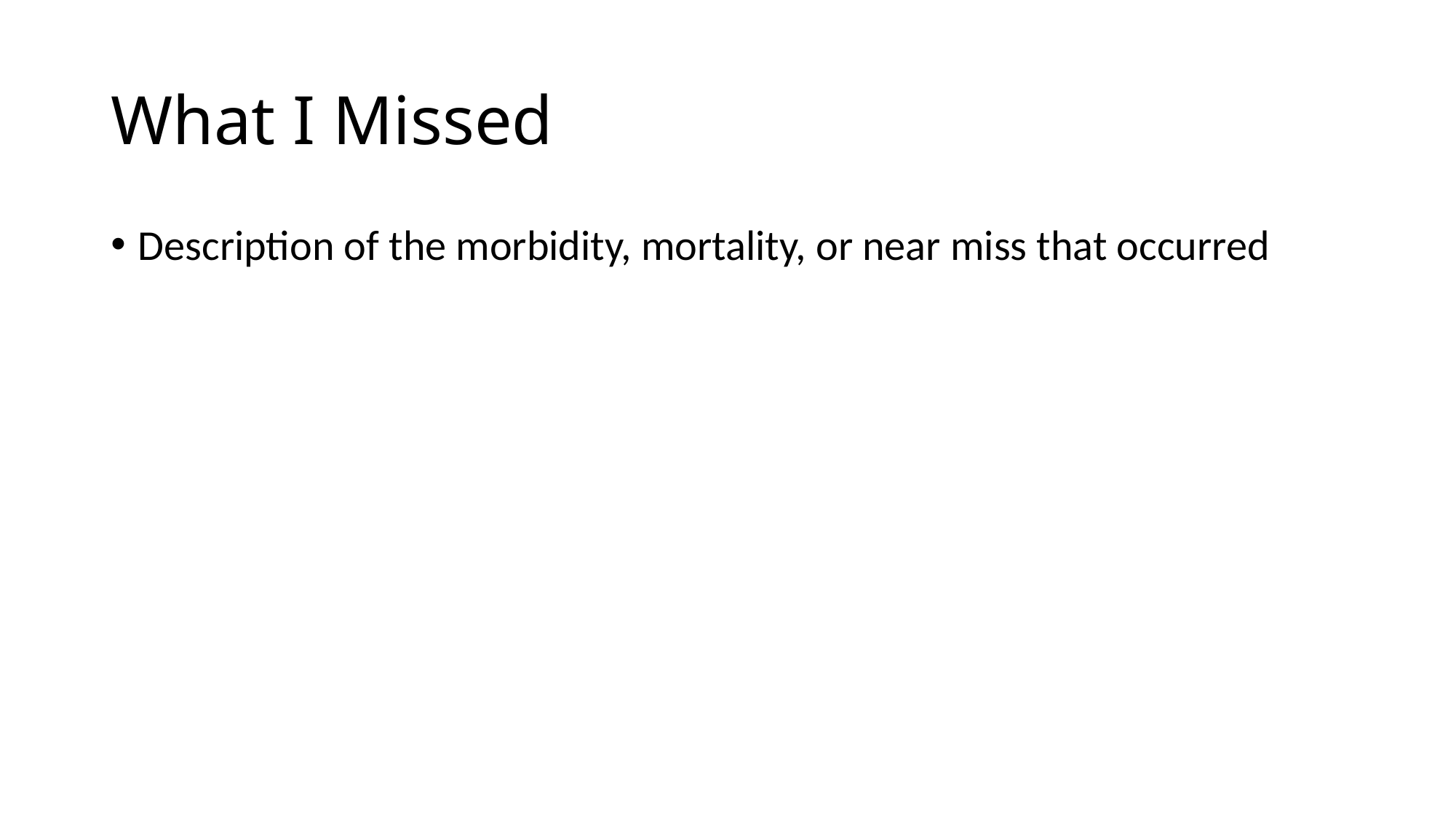

# What I Missed
Description of the morbidity, mortality, or near miss that occurred

## Slide 12
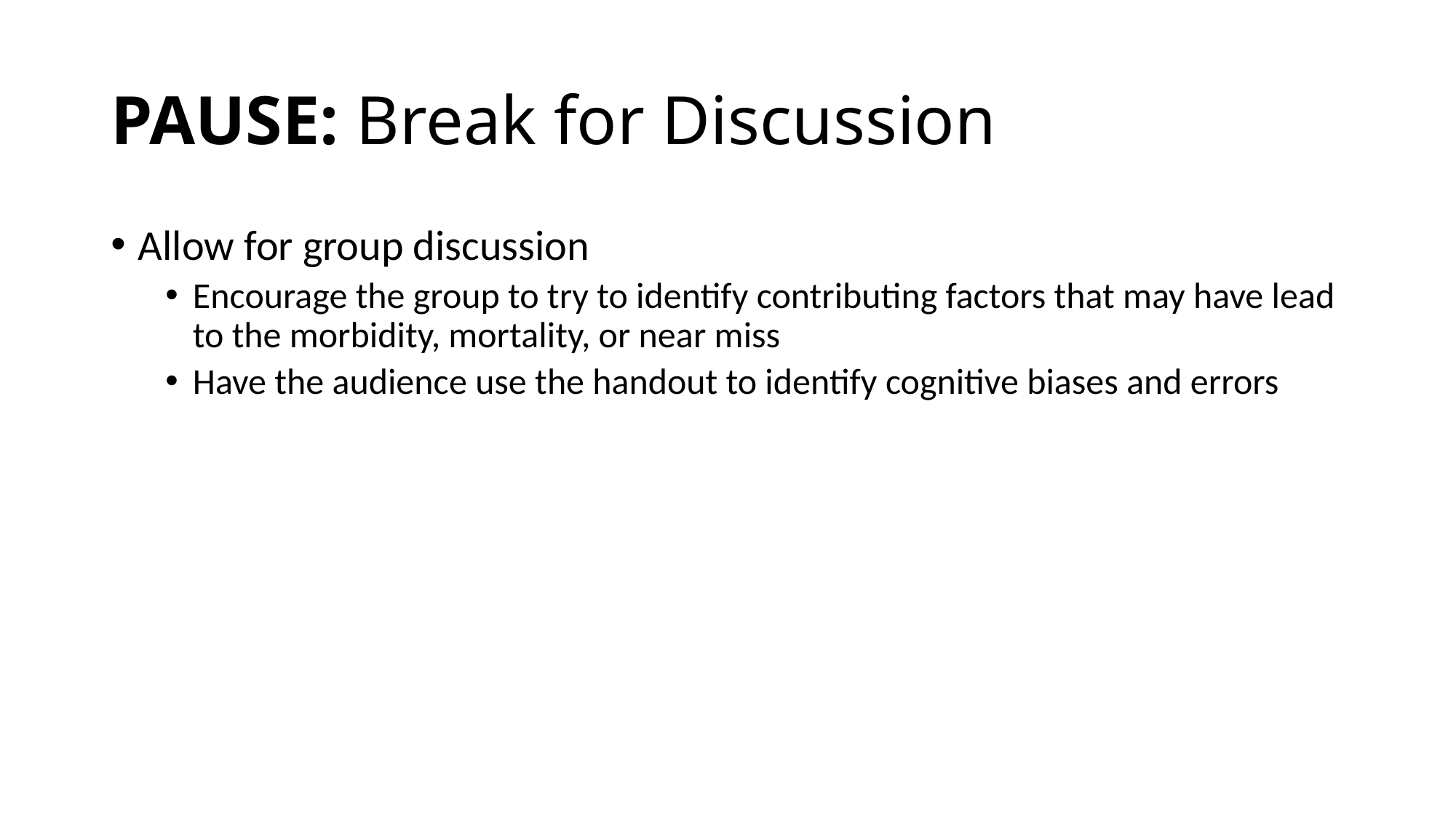

# PAUSE: Break for Discussion
Allow for group discussion
Encourage the group to try to identify contributing factors that may have lead to the morbidity, mortality, or near miss
Have the audience use the handout to identify cognitive biases and errors

## Slide 13
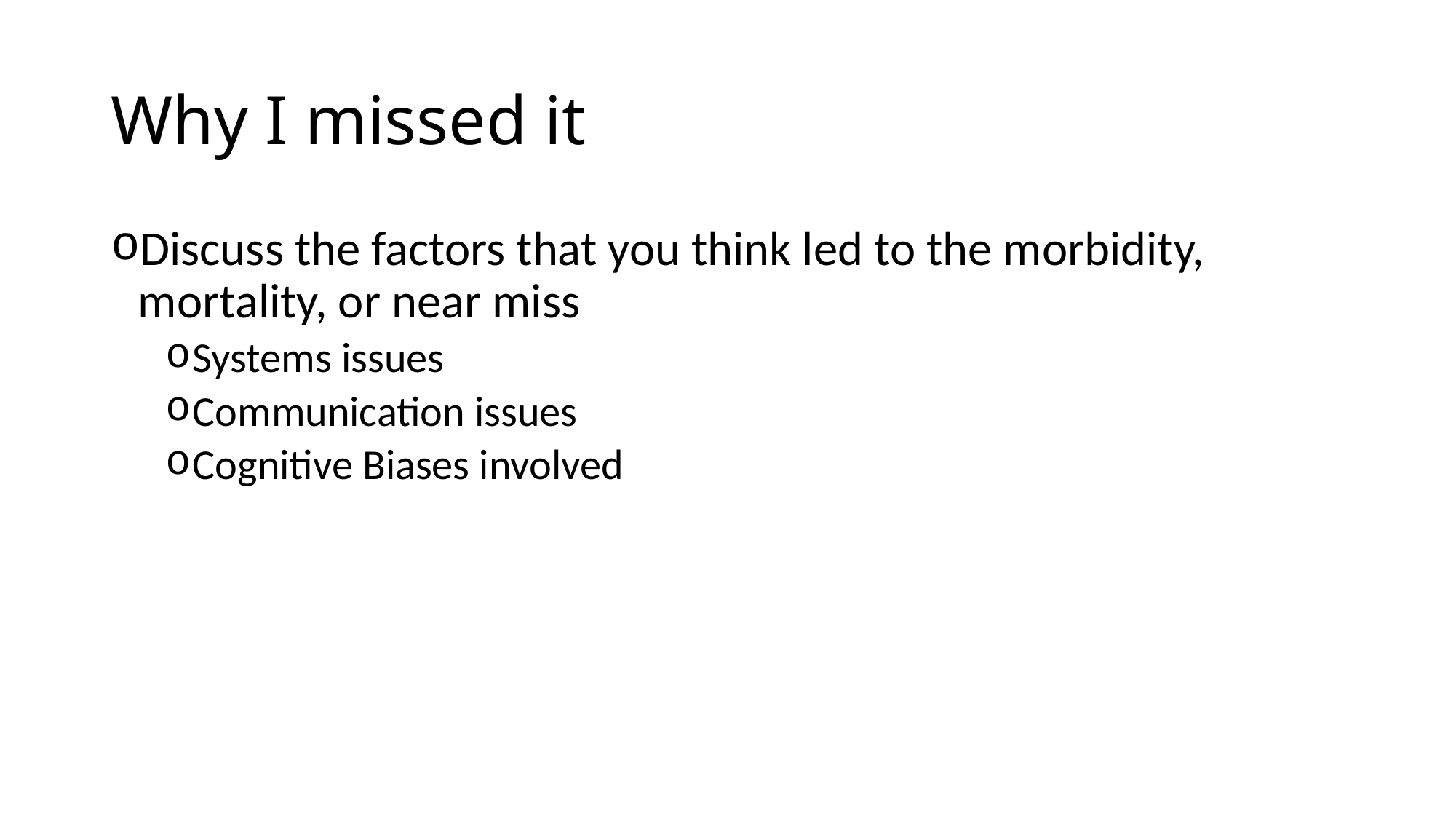

# Why I missed it
Discuss the factors that you think led to the morbidity, mortality, or near miss
Systems issues
Communication issues
Cognitive Biases involved

## Slide 14
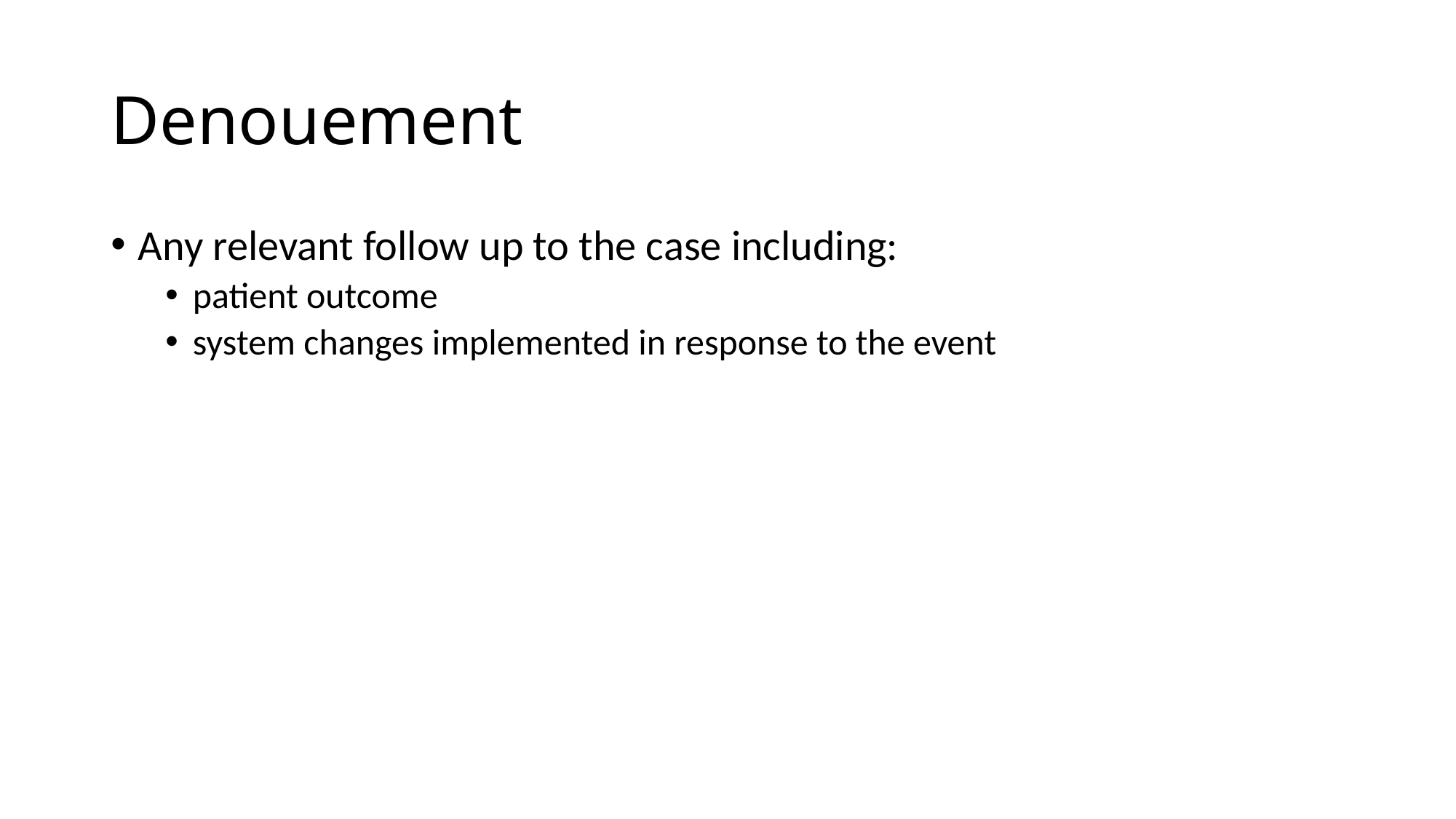

# Denouement
Any relevant follow up to the case including:
patient outcome
system changes implemented in response to the event

## Slide 15
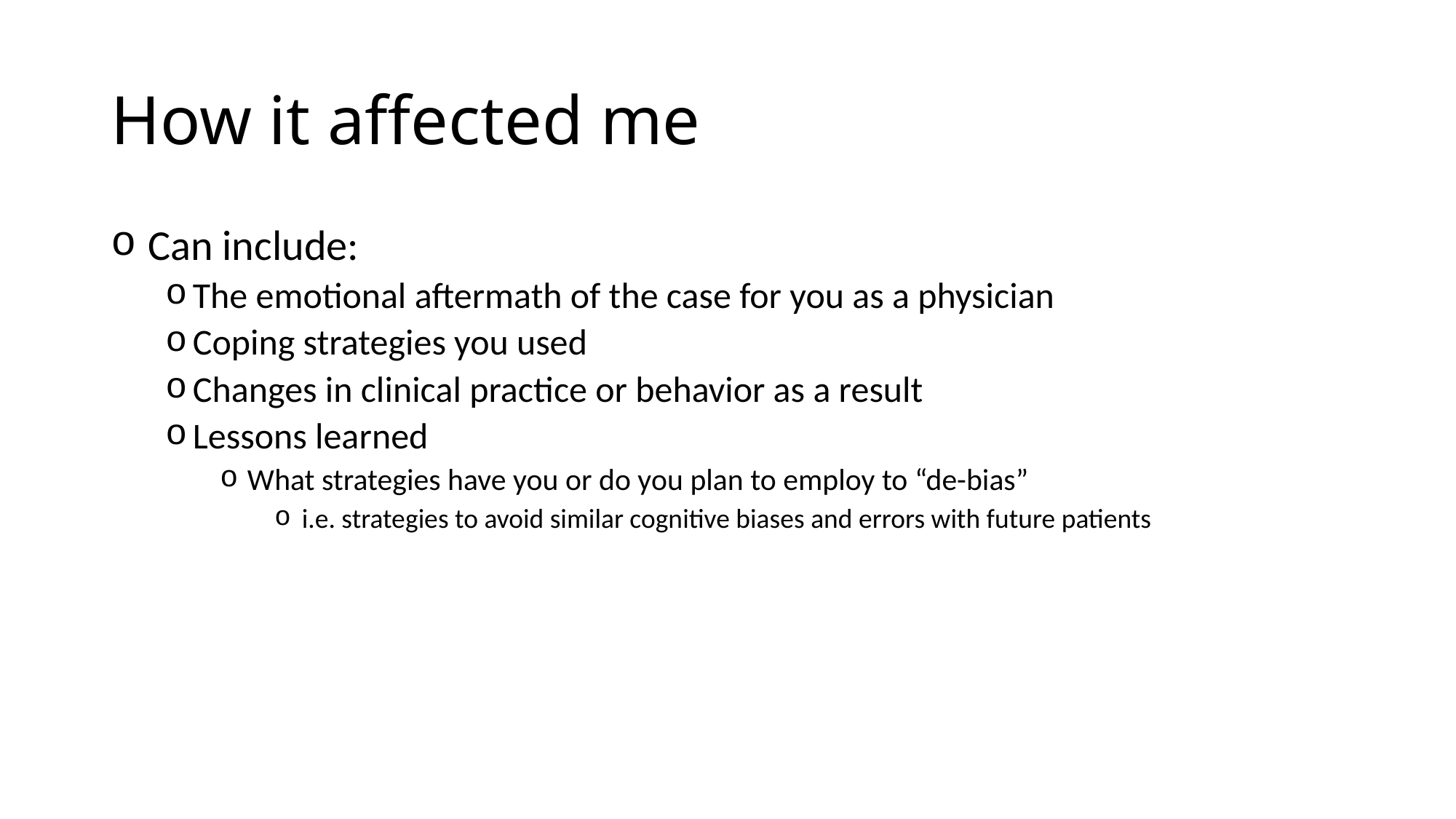

# How it affected me
 Can include:
The emotional aftermath of the case for you as a physician
Coping strategies you used
Changes in clinical practice or behavior as a result
Lessons learned
What strategies have you or do you plan to employ to “de-bias”
i.e. strategies to avoid similar cognitive biases and errors with future patients

## Slide 16
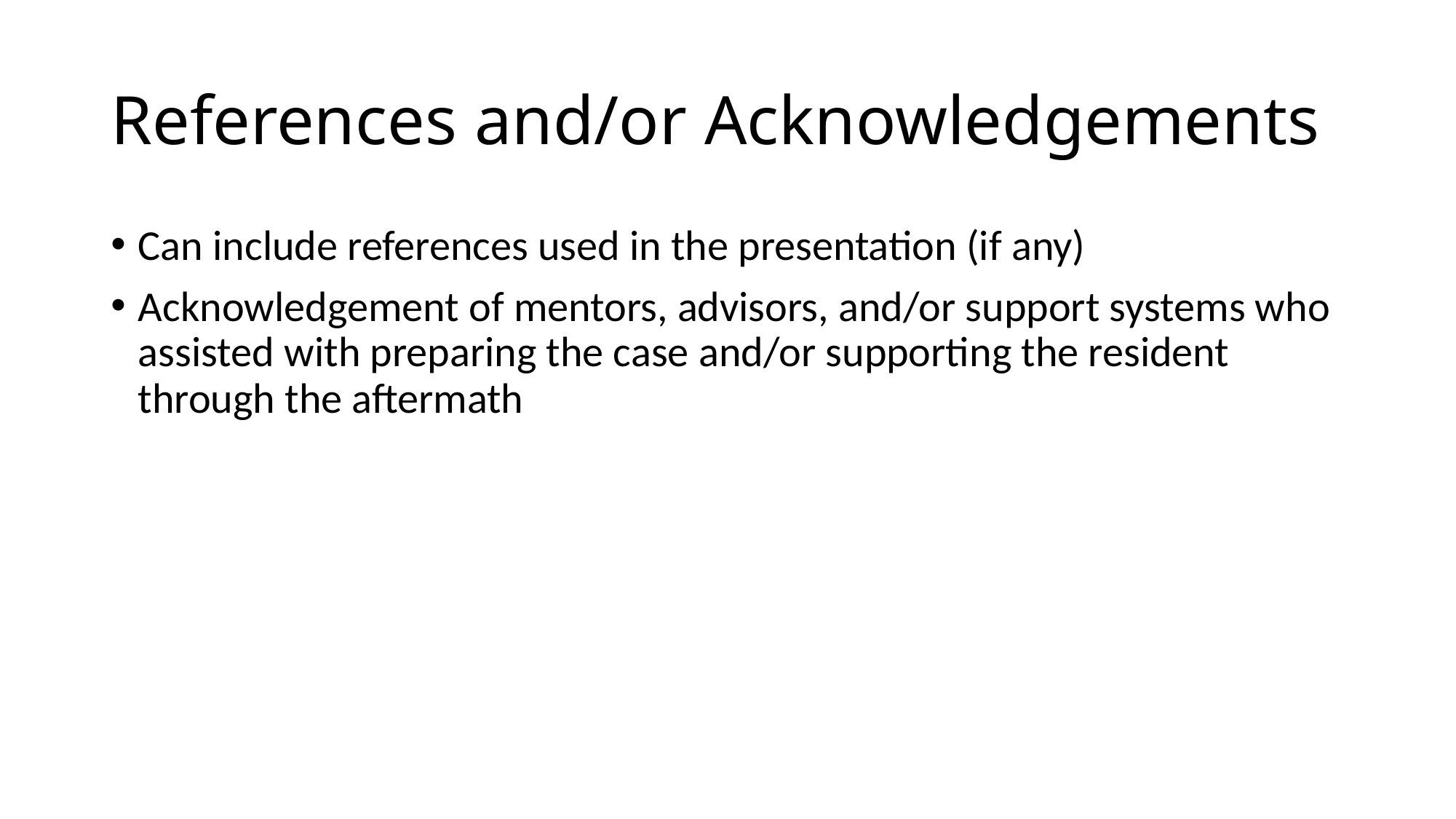

# References and/or Acknowledgements
Can include references used in the presentation (if any)
Acknowledgement of mentors, advisors, and/or support systems who assisted with preparing the case and/or supporting the resident through the aftermath

## Slide 17
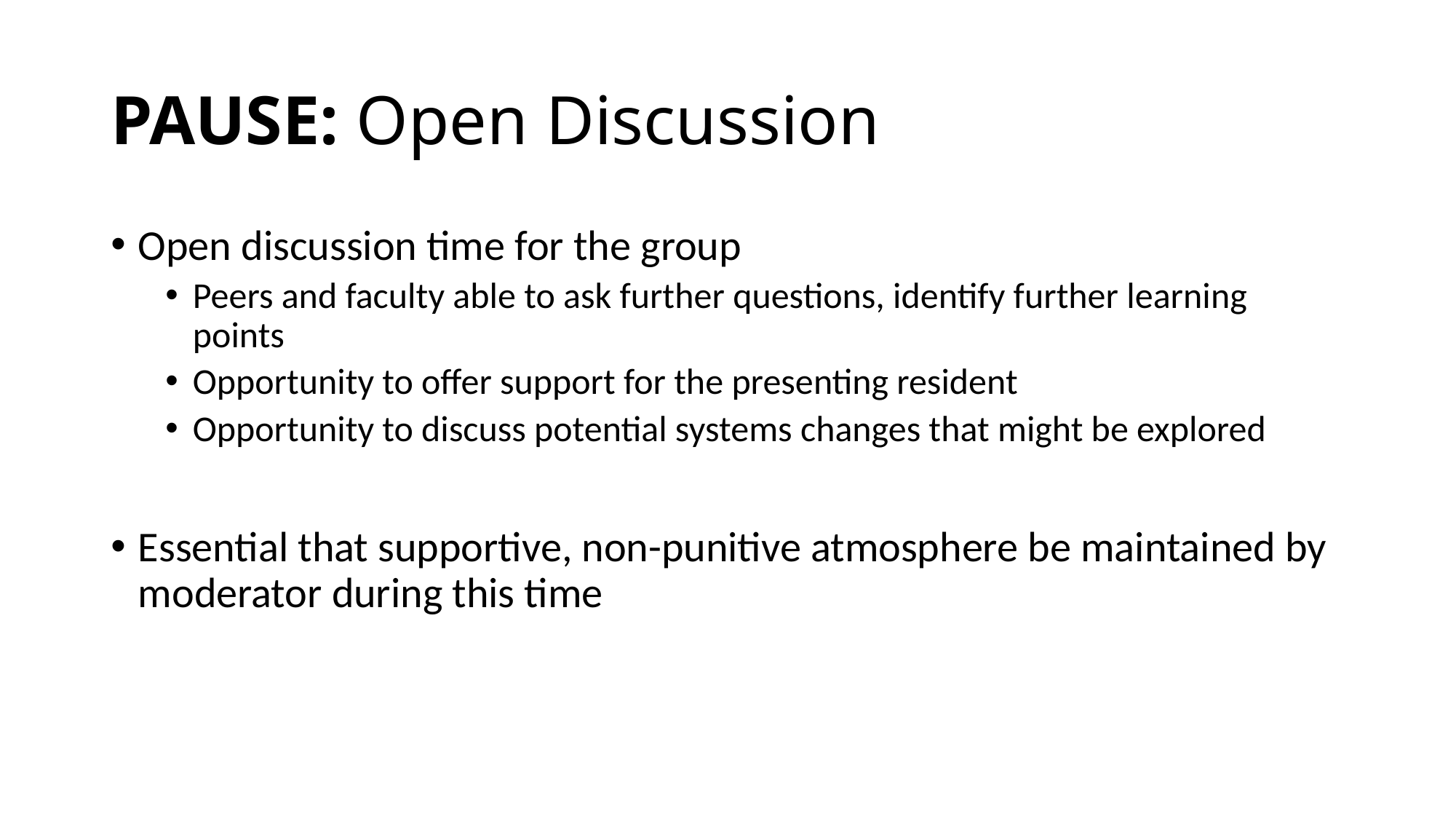

# PAUSE: Open Discussion
Open discussion time for the group
Peers and faculty able to ask further questions, identify further learning points
Opportunity to offer support for the presenting resident
Opportunity to discuss potential systems changes that might be explored
Essential that supportive, non-punitive atmosphere be maintained by moderator during this time
