# Supplementary material for: A Resident Morbidity and Mortality Conference Curriculum to Teach Identification of Cognitive Biases, Errors, and Debiasing Strategies
Source: MedEdPORTAL. 2021 Oct 28;17:11190. doi: 10.15766/mep_2374-8265.11190 (PMC8551265; doi:10.15766/mep_2374-8265.11190)
Supplement: Supplementary file 1 — M&M Resident Presenter Guide.docxM&M Advisors Guide.docxM&M Introduction and Template.pptxM&M Discussion Handout.docx [file mep_2374-8265.11190-s001.zip › Educational_Summary_Report_11190.pdf]

MedEdPORTAL® | The AAMC Journal of Teaching and Learning Resources

Original Publication Open Access

A Resident Morbidity and Mortality Conference Curriculum to Teach Identification of Cognitive Biases, Errors, and Debiasing Strategies

Anne Whitehead, MD*

*Corresponding author: annewhit@iu.edu

Abstract

Introduction: The morbidity and mortality (M&M) conference has long been a part of the education of residents of all specialties in the United States, yet its structure is variable across training programs. Recent literature has described the use of M&M as a forum for education in quality improvement methodology; however, a structure focusing on education in cognitive biases and errors has not been previously described in *MedEdPORTAL*. **Methods:** This structured M&M conference series called upon resident presenters and peers in the audience to examine cognitive biases and errors involved in specific patient cases. Associated materials included preparatory guidelines provided to faculty advisors and resident presenters, a presentation template used during the introductory session, and a handout used during the discussion portions of presentations. **Results:** During the 2019-2020 academic year, a total of 24 PGY 2 pediatrics residents presented M&M cases. They identified a mean of 3.7 ($SD = 1.9$) cognitive biases and/or errors per case and a mean of 1.7 ($SD = 0.7$) debiasing strategies per case. Peers in the audience were also successful in identifying potential biases and errors at play during presentations. **Discussion:** We found that through this M&M conference structure, residents were able to demonstrate the ability to identify cognitive errors and biases both within themselves and in peers. This provided an effective forum for the identification and discussion of debiasing strategies, even when the series was forced to transition to a virtual format due to the COVID-19 pandemic.

Keywords
Morbidity, Mortality, Cognitive Bias, Cognitive Error, Quality Improvement, Patient Safety, Clinical Reasoning, Diagnostic Reasoning, Case-Based Learning

Educational Objectives

By the end of this activity, learners will be able to:

1. Describe the importance of identifying and correcting cognitive biases and errors in improving patient safety.
2. Identify cognitive biases and errors that have affected their own medical decision-making care of patients.
3. Formulate personal debiasing strategies for their own practice of medicine.

Introduction

Morbidity and mortality (M&M) conferences have long been a part of all residencies, as required by the ACGME.[1] One of the primary goals of an M&M conference is resident education in patient safety, and a wide variety of conference structures have been proposed and implemented to try to meet this objective.[2-4]

Citation:
Whitehead A. A resident morbidity and mortality conference curriculum to teach identification of cognitive biases, errors, and debiasing strategies. *MedEdPORTAL*. 2021;17:11190.
https://doi.org/10.15766/mep_2374-8265.11190

An understanding of cognitive biases and errors is a vital component of improving patient safety and reducing diagnostic error.[5] Cognitive errors account for a large portion of medical errors, deaths, and malpractice claims, yet this subject is often underrepresented in patient safety education.[6,7]

There are several examples of the use of the M&M conference as an opportunity to teach and practice patient safety concepts and quality improvement (QI) in *MedEdPORTAL* and beyond. The focus in the existing literature on the use of M&M for patient safety education is largely on the identification and remedy of systems-based errors and education in QI methodology.[3,8-11] Some previously described conference structures do encourage residents to briefly identify any cognitive biases and/or errors,[2,9] but in these cases, this was not identified as a primary educational objective.

Copyright © 2021 Whitehead. This is an open-access publication distributed under the terms of the Creative Commons Attribution license.

A small number of curricula primarily aimed at teaching the identification and prevention of cognitive errors currently exist in *MedEdPORTAL*. These take a variety of formats, including workshop,[12,13] simulation,[14,15] case-based discussion,[16] video,[17] and lecture.[13,18] While the majority of these curricula use patient cases to help learners meet the objectives, only one encourages learners to reflect on a real case in which they were involved.[18] In that curriculum, the self-reflection is only a small portion of the activity and is not followed up by any discussion.

We restructured our existing M&M curriculum for pediatrics residents to focus on identification of cognitive biases and errors and, subsequently, debiasing strategies. We emphasized identification of cognitive errors and debiasing strategies as a primary learning objective and structured the conference to include respectful audience discussion to further practice identification of cognitive errors and biases. We required that residents present a case in which they were involved to encourage self-reflection and self-awareness, which are important emphases in pediatrics residency milestones pertaining to professionalism and practice-based learning.[19] Our intended primary learners for this conference series were the pediatric resident presenters, with the audience comprising residents, students, and a small number of faculty as the secondary learners.

Methods

Conference Scheduling

M&M conferences were 1 hour in length and were held 13 times during the 2019-2020 academic year during pediatric residency noon conference. Pediatric chief residents assigned every categorical pediatrics resident in their second year of residency a presentation date at the beginning of the academic year. One, two, or three residents presented at each conference, except during the introductory session, at which only the faculty advisor presented. A total of 24 PGY 2 pediatrics residents were scheduled to present during these sessions.

Preparation for Resident Presenters

At the beginning of the academic year, chief residents distributed a written set of expectations (Appendix A) and two articles regarding cognitive error and bias to help the second-year residents prepare for their presentations.[20,21] The written expectations prompted the residents to identify a case and provide a synopsis at least 1 month prior to presentation, which the faculty advisor and pediatric chief residents then reviewed. The advisor used guidelines laid out in the advisor's guide (Appendix B) to help the resident determine if their chosen case was appropriate for meeting the goals of the M&M conference. The resident expectations required that residents choose a case in which they were actively involved in the care of the patient at the time error(s) occurred leading to morbidity, mortality, or a near miss. The expectations encouraged resident presenters to identify and work with a faculty mentor who was also involved in the care of the patient. Residents who had difficulty identifying an appropriate case and/or mentor could ask for help from the chief residents and/or the faculty advisor.

Residents used a template (Appendix C) to prepare a presentation of 15-20 minutes in length. They were instructed to use the articles[20,21] they were given to aid in the identification of cognitive errors that may have or did contribute to the morbidity, mortality, or near miss and to include any identified biases and errors in their presentation. The faculty advisor reviewed the presentation slides roughly 1 week prior to the scheduled presentation date and gave feedback to ensure each presentation was in keeping with the expectations set forth in the advisor guide (Appendix B).

Preparation for Chief Residents and Faculty Advisor

The faculty advisor, who had served in the role of faculty advisor to M&M for 2 years prior to the implementation of this specific conference structure, met with the three chief residents prior to the start of the academic year to discuss the structure and intended tone of the conference, the objectives of the structure, and strategies for facilitating the conference. All four had access to all materials available in Appendices A-D as well as to the two articles provided to all resident presenters.[20,21]

Conference Structure and Facilitation

The same faculty advisor attended and moderated every M&M conference session along with at least one of the three pediatric chief residents. Pediatrics residents and other residents rotating in the children's hospital, as well as medical students rotating through the school of medicine, attended the sessions. Faculty in program leadership, as well as faculty who were involved as faculty mentors for the cases presented, also often attended. Conferences were not open for attendance by anyone from outside the institution. Sessions had variable attendance over the course of the year but ranged between 10 and 50 audience members, and most attendees were resident peers.

The written expectations (Appendix A) and presentation template (Appendix C) prompted the residents to pause their presentations after sharing the details of the case to allow for audience discussion. Chief residents and/or the faculty advisor could also prompt the presenter to pause for discussion if they did not do

Copyright © 2021 Whitehead. This is an open-access publication distributed under the terms of the Creative Commons Attribution license.

so themselves. The chief resident in attendance then distributed a handout comprising a list of cognitive biases summarized from existing literature[20,22] (Appendix D) to audience members and asked them to identify potential biases at play in the case. The resident presenters and/or chief residents facilitated the ensuing discussion, depending on the preference of the presenter. Following this discussion, the resident presenters presented their self-identified biases. They then identified lessons learned from the case and were encouraged, but not required, to discuss strategies to overcome the biases they identified. After the resident presenter finished, an open audience discussion ensued.

Establishing the Nonpunitive, Nonjudgmental Tone

The first M&M session took place in July at the beginning of the 2019-2020 academic year. It served as an introduction to the conference series. The faculty advisor for the series presented expectations and ground rules, emphasizing the nonpunitive and private nature of the conference series. She then presented an example case in the same format requested of the resident presenters (Appendices A-C). The advisor chose the case of a patient she had cared for as an attending physician that involved significant morbidity. The faculty advisor solicited audience input and identified her own cognitive biases and errors in the same manner expected of resident presenters. While this did serve as an example for case structure and presentation format, it was of specific importance that the case presented at this session was one the faculty attended as a physician. With this structure, we aimed to model self-reflective behavior and to decrease the vulnerability associated with the inherent hierarchical nature within medical education, which at times can be counterproductive to open discussion.

Adaptation to COVID-19

With COVID-19 becoming widespread in the United States in March 2020, all subsequent residency noon conferences, including M&M, transitioned to a virtual format via secure Zoom meetings. An institutional Zoom account was used to set up password-protected conferences for all remote resident education sessions during this time, and this same process was used for M&M conferences. Residents presented from the previously used conference room with a very small in-person audience or offsite remotely, depending on personal choice and/or circumstance. Discussion was fostered by both the audiovisual features of Zoom and the chat function, which was monitored by one of the pediatric chief residents. Because medical students were removed from clerkships during this time period, no medical students attended during the virtual sessions. During the 2019-2020 academic year, eight in-person sessions took place, and five virtual sessions took place after the start of the COVID-19 pandemic.

Evaluation

Twenty-four residents presented M&M cases during the series. During each of the 12 resident presentation sessions, the faculty advisor kept a record of the cognitive errors identified by the residents presenting their cases, as well as those identified during audience discussion. She recorded the cognitive errors on a Microsoft Excel spreadsheet in real time during the conferences. The faculty advisor also recorded resident presenter self-identified strategies for cognitive debiasing and error reduction during each session. Additionally, she reviewed the presentation slides to ensure no self-identified biases or debiasing strategies were missed. She recorded the cognitive biases identified verbally by audience members during the two pauses for discussion in each presentation. After the transition to virtual conferences in response to COVID-19, the faculty advisor recorded errors and debiasing strategies discussed verbally as well as those written in the chat function.

No information identifying the resident presenter, audience members, or patient discussed was included in this record. The Indiana University Institutional Review Board determined that this intervention was exempt from review.

We analyzed the data with descriptive statistics using Microsoft Excel. The faculty advisor grouped debiasing strategies identified by residents into categories based on those described by Croskerry in the first table in his article "The Importance of Cognitive Errors in Diagnosis and Strategies to Minimize Them."[21]

Results

All 24 resident presenters were successful in self-identifying cognitive biases and in identifying biases contained in cases presented by their peers. Resident presenters were also able to identify a variety of debiasing strategies and to discuss methods of implementation.

Self-Identification of Cognitive Bias and Errors

Residents identified a mean of 3.7 ($SD = 1.9$) cognitive biases per case, with a range of one to eight. Presenters collectively self-identified 27 distinct errors and biases using the reference materials provided. The most common errors and biases identified were anchoring bias (12 cases), omission bias (eight cases), and framing effect (seven cases; see Figure). Eleven of 24 residents identified knowledge gaps and/or deficits as contributing factors in their cases.

Copyright © 2021 Whitehead. This is an open-access publication distributed under the terms of the Creative Commons Attribution license.

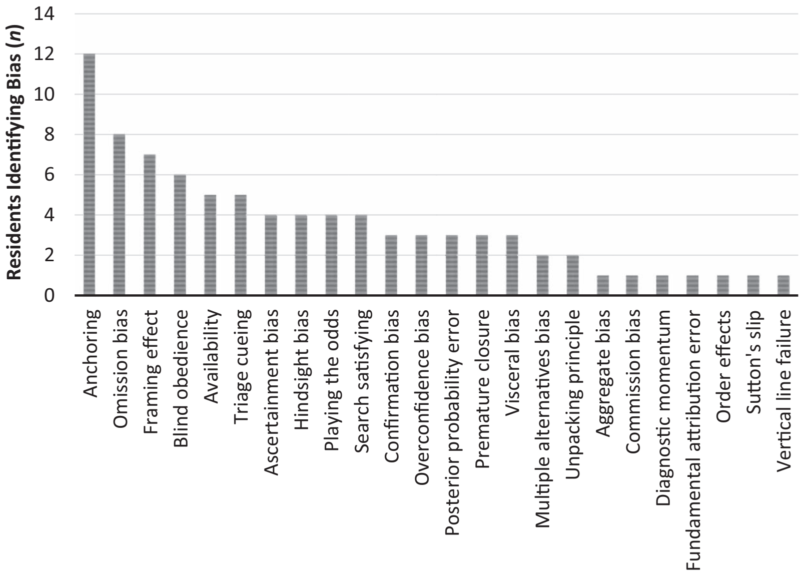

Figure. Number of residents self-identifying specific bias in their cases.

Peer Identification of Cognitive Bias

Biases identified by peers in the audience were similar, but not identical, to those identified by the presenters, with anchoring bias, framing effect, and blind obedience identified most often. Notably, in one case, an audience member identified racial bias as a factor that had not been identified previously for the case.

Debiasing Strategies Identified

Resident presenters identified a mean of 1.7 ($SD = 0.7$) debiasing strategies per presentation, with a range of one to three. Several proposed strategies involved development of insight and self-awareness. A sample of some, but not all, of the strategies identified by residents is included in the Table.

Discussion

Educational Value

We successfully integrated teaching on cognitive errors and biases into a commonly used M&M framework. The M&M conference was particularly well suited to explore personal vulnerability to cognitive biases and error as residents were able to reflect on cases and decisions in which they were involved personally. While this served to educate residents on the human factors that could affect patient safety, it also supported residents in meeting professionalism and practice-based learning milestones, which allowed the learners to develop skills in self-reflection and making change in response to that reflection.

We implemented this conference series with pediatric residents, but it could be easily applied with residents in any specialty training program. Cognitive biases and errors are important factors in medical error and patient safety regardless of specialty and—as the review of the literature suggested—underrepresented in education related to patient safety. The M&M conference already occurs throughout postgraduate training programs, so this format could be easily used as a part of, or as a replacement for, an existing conference series.

We also learned that the conference structure could be translated into a virtual format. Audience participants continued to engage and identify cognitive biases and errors despite the change in format. This has been of particular importance during the COVID-19 pandemic. It may also contribute to increased accessibility of the conference series for trainees and faculty in the future, even when the need for social distancing is not present.

Potential Barriers

In many programs and at many institutions, it is not the typical practice to have residents discuss cases in which they were actively involved during M&M, as we required in our conference format. The format focused on learning from the more individual and subjective experience of the care team, rather than the more objective and detached approach that a third party might take. This may then be met with resistance from presenters, as vulnerability is increased when discussing one's own patients and decision-making. This resistance could likely be countered somewhat by the vulnerability modeled in the faculty advisor M&M presentation. Diligent oversight of resident case selection and presentation by the faculty advisor would be especially

Copyright © 2021 Whitehead. This is an open-access publication distributed under the terms of the Creative Commons Attribution license.

Table. Examples of Debiasing Strategies Identified and Implemented by Resident Presenters

Category of Debiasing Strategy	Examples of Debiasing Strategies Proposed and/or Implemented by Residents
Reflecting, developing insight.	"I explored the reasons behind my personal tendency toward inaction."
Decreasing reliance on memory.	"I make sure I know where all my equipment is before I go to a delivery."
	"I always use the order set when ordering medications less familiar to me."
Improving training/knowledge base.	"I researched the protocol around changing a fresh feeding tube."
Using cognitive forcing strategies.	"I treat transferred patients like brand new patients."
	"I force myself to pay closer attention in nonverbal patients."
Increasing reliance on objective data.	"I use tools to visualize data trends in the electronic medical record."
	"I go back to the bedside to reexamine before making changes."

important in preparing residents unaccustomed to discussing their own errors and biases in such a format.

Because of the narrower focus of the conference structure mainly on cognitive bias, this M&M series would not serve as the only educational element on patient safety within a residency curriculum. It would be very important to supplement it with specific teaching on systems issues, systems solutions, and QI methodology in a different portion of the patient safety curriculum. This may be a potential barrier in programs that already rely heavily on M&M conferences for these patient safety topics.

Lessons Learned

The team encountered few barriers in implementing this adjusted M&M curriculum. We anticipated residents might express hesitation in sharing their personal biases with the audience and with allowing the audience to discuss the biases they identified. For this reason, we left the option open for residents to focus only on systems-based issues in the case by simply proposing a QI intervention. Despite this option, every resident presenter chose to self-identify biases, and 22 of the 24 chose to open the discussion of biases to the group.

This willingness to engage in this level of reflection and discussion was likely multifactorial. M&M had been a requirement for second-year residents in the program for years prior to implementing this specific structure, and a nonpunitive tone was already well established. We also suspect that having an attending M&M share openly with the group at the beginning of the series was another important element in establishing tone.

This conference structure required resident presenters to be open and vulnerable during their presentations. It also required commitment from the audience, especially faculty members in attendance, to establish and maintain the appropriate supportive tone. This was already well established in our program, but in a program with a more punitive M&M culture, faculty expectation-setting would be essential. It would be prudent to identify faculty champions for this approach who would, in conjunction with the faculty advisor, redirect and enforce the nonpunitive and supportive tone in the audience, especially if faculty less familiar with the objectives of the conference were in attendance. In our sessions, residency program leadership in attendance often filled this role.

While residents were able to identify a variety of cognitive biases in the cases they presented, it was notable that there were some bias types—such as anchoring and omission biases—that were identified much more often than others. It may be that these types of bias truly do occur more frequently in resident patient care and therefore should be addressed with further educational intervention around debiasing strategies specific to these bias types. It is also possible, though, that these types of bias are easier for residents to self-identify and admit to than others. Biases such as visceral or racial biases may be viewed as less socially acceptable to experience, and therefore, residents may be consciously or subconsciously less likely to identify them as factors in a case. It will be important moving forward to explore these possibilities and work to further develop a culture of openness surrounding M&M to allow for safe discussion of these potentially more uncomfortable biases.

Each presentation included a space for open discussion, which proved to be valuable. There was no specific structure used or expectation set for this time. Both residents and faculty members helped shape the discussion depending on the details of the case presented. We found that this open discussion often focused either on ideas for improving future care or on supporting the resident presenter. This latter theme seemed to emerge often when residents exhibited or expressed some degree of distress about the case presented, and several residents indicated that the experience of receiving this support during the open discussion was helpful.

Limitations

The evaluation of our intervention was limited to observations made during the session. Data collection and analysis were done

Copyright © 2021 Whitehead. This is an open-access publication distributed under the terms of the Creative Commons Attribution license.

by the faculty advisor, who was also involved in moderating the M&M session. Future evaluation of this conference structure might be more robust with an uninvolved third party collecting data. Residents did not undergo a presession assessment, so we could not quantify the degree of improvement, if any, in identifying cognitive bias and error. The data collected did not allow us to determine if the skills demonstrated in identifying cognitive errors and biases in preparation for and during the session translated to residents identifying biases occurring in real time as they cared for patients. Similarly, while residents showed they could identify debiasing strategies, we did not evaluate whether they implemented these strategies, how effectively they did so, or the impact on patient safety.

Further evaluation of the impact of this conference series might include surveys to evaluate resident comfort with identifying bias and debiasing strategies, faculty perception of resident ability to avoid bias, and most importantly—though perhaps most difficult to study—frequency and/or severity of patient safety concerns following the educational intervention.

Appendices

A. M&M Resident Presenter Guide.docx

B. M&M Advisors Guide.docx

C. M&M Introduction and Template.pptx

D. M&M Discussion Handout.docx

All appendices are peer reviewed as integral parts of the Original Publication.

Anne Whitehead, MD: Assistant Professor of Emergency Medicine and Pediatrics, Department of Emergency Medicine, Indiana University School of Medicine; ORCID: https://orcid.org/0000-0002-8392-2988

Disclosures
None to report.

Funding/Support
None to report.

Ethical Approval
The Indiana University Institutional Review Board deemed further review of this project not necessary.

References

1. Accreditation Council for Graduate Medical Education. *The Program Directors' Guide to the Common Program Requirements (Residency)*. Accreditation Council for Graduate Medical Education; 2020. Accessed December 2, 2020. https://www.acgme.org/Portals/0/PFAssets/ProgramResources/PDGuideResidency.pdf
2. Cifra CL, Miller MR. Envisioning the future morbidity and mortality conference: a vehicle for systems change. *Pediatr Qual Saf*. 2016;1(2):e003. https://doi.org/10.1097/pq9.0000000000000003
3. Garcia C, Goolsarran N. Learning from errors: curriculum guide for the morbidity and mortality conference with a focus on patient safety concepts. *MedEdPORTAL*. 2016;12:10462. https://doi.org/10.15766/mep_2374-8265.10462
4. Orlander JD, Barber TW, Fincke BG. The morbidity and mortality conference: the delicate nature of learning from error. *Acad Med*. 2002;77(10):1001-1006. https://doi.org/10.1097/00001888-200210000-00011
5. Royce CS, Hayes MM, Schwartzstein RM. Teaching critical thinking: a case for instruction in cognitive biases to reduce diagnostic errors and improve patient safety. *Acad Med*. 2019; 94(2):187-194. https://doi.org/10.1097/ACM.0000000000002518
6. Saposnik G, Redelmeier D, Ruff CC, Tobler PN. Cognitive biases associated with medical decisions: a systematic review. *BMC Med Inform Decis Mak*. 2016;16:138. https://doi.org/10.1186/s12911-016-0377-1
7. Wachter RM. Why diagnostic errors don't get any respect—and what can be done about them. *Health Aff (Millwood)*. 2010;29(9): 1605-1610. https://doi.org/10.1377/hlthaff.2009.0513
8. Bechtold ML, Scott S, Nelson K, Cox KR, Dellsperger KC, Hall LW. Educational quality improvement report: outcomes from a revised morbidity and mortality format that emphasised patient safety. *Qual Saf Health Care*. 2007;16(6):422-427. Article republished in: *Postgrad Med J*. 2008;84(990):211-216. https://doi.org/10.1136/qshc.2006.021139
9. Calder LA, Kwok ESH, Cwinn AA, et al. Enhancing the quality of morbidity and mortality rounds: the Ottawa M&M model. *Acad Emerg Med*. 2014;21(3):314-321. https://doi.org/10.1111/acem.12330
10. Walker M, Rubio D, Horstman M, Trautner B, Stewart D. Stop the blame game: restructuring morbidity and mortality conferences to teach patient safety and quality improvement to residents. *MedEdPORTAL*. 2016;12:10475. https://doi.org/10.15766/mep_2374-8265.10475
11. Werner JA. An integrated, multimodal resident curriculum in patient safety and quality improvement. *MedEdPORTAL*. 2017; 13:10641. https://doi.org/10.15766/mep_2374-8265.10641
12. Daniel M, Carney M, Khandelwal S, et al. Cognitive debiasing strategies: a faculty development workshop for clinical teachers

Copyright © 2021 Whitehead. This is an open-access publication distributed under the terms of the Creative Commons Attribution license.

in emergency medicine. *MedEdPORTAL*. 2017;13:10646. https://doi.org/10.15766/mep_2374-8265.10646

13. Hunzeker A, Amin R. Teaching cognitive bias in a hurry: single-session workshop approach for psychiatry residents and students. *MedEdPORTAL*. 2016;12:10451. https://doi.org/10.15766/mep_2374-8265.10451
14. Paolo WF. Medical heuristics and cognitive decision errors: modeling thought processes through an integrative curriculum. *MedEdPORTAL*. 2013;9:9375. https://doi.org/10.15766/mep_2374-8265.9375
15. Weil J, Cassara M. Occult sepsis masked by trauma—exploration of cognitive biases through simulation with emergency medicine residents. *MedEdPORTAL*. 2020;16:11023. https://doi.org/10.15766/mep_2374-8265.11023
16. Chew KS, van Merrienboer J, Durning S. Teaching cognitive biases in clinical decision making: a case-based discussion. *MedEdPORTAL*. 2015;11:10138. https://doi.org/10.15766/mep_2374-8265.10138
17. Stiegler M, Goldhaber-Fiebert S. Understanding and preventing cognitive errors in healthcare. *MedEdPORTAL*. 2015;11:10000. https://doi.org/10.15766/mep_2374-8265.10000
18. Ruedinger E, Mathews B, Olson A. Decision — diagnosis: an introduction to diagnostic error and medical decision-making. *MedEdPORTAL*. 2016;12:10378. https://doi.org/10.15766/mep_2374-8265.10378
19. Accreditation Council for Graduate Medical Education. *Pediatrics Milestones*. Accreditation Council for Graduate Medical Education; 2020. Accessed December 2, 2020. https://www.acgme.org/Portals/0/PDFs/Milestones/PediatricsMilestones.pdf?ver=2017-07-24-124802-340
20. Croskerry P. Achieving quality in clinical decision making: cognitive strategies and detection of bias. *Acad Emerg Med*. 2002;9(11):1184-1204. https://doi.org/10.1197/aemj.9.11.1184
21. Croskerry P. The importance of cognitive errors in diagnosis and strategies to minimize them. *Acad Med*. 2003;78(8):775-780. https://doi.org/10.1097/00001888-200308000-00003
22. Ogdie AR, Reilly JB, Pang WG, et al. Seen through their eyes: residents' reflections on the cognitive and contextual components of diagnostic errors in medicine. *Acad Med*. 2012;87(10):1361-1367. https://doi.org/10.1097/ACM.0b013e31826742c9

Received: January 6, 2021
Accepted: August 1, 2021
Published: October 28, 2021

Copyright © 2021 Whitehead. This is an open-access publication distributed under the terms of the Creative Commons Attribution license.
